# Synthetic lethality in *CCNE1*-amplified high grade serous ovarian cancer through combined inhibition of Polo-like kinase 1 and microtubule dynamics

**DOI:** 10.18632/oncotarget.25386

**Published:** 2018-05-25

**Authors:** Sabrina Noack, Monika Raab, Yves Matthess, Mourad Sanhaji, Andrea Krämer, Balázs Győrffy, Lars Kaderali, Ahmed El-Balat, Sven Becker, Klaus Strebhardt

**Affiliations:** ^1^ Department of Gynecology, Goethe-University, Frankfurt am Main, Germany; ^2^ German Cancer Consortium DKTK, German Cancer Research Center, Heidelberg, Germany; ^3^ MTA TTK Lendület Cancer Biomarker Research Group, Institute of Enzymology, Hungarian Academy of Sciences, Budapest, Hungary; ^4^ Institute of Bioinformatics, University Medicine Greifswald, Greifswald, Germany; ^5^ Semmelweis University 2nd Department of Pediatrics, Budapest, Hungary

**Keywords:** ovarian cancer, protein kinases, cell cycle, paclitaxel, sensitization

## Abstract

The taxanes are effective microtubule-stabilizing chemotherapy drugs that inhibit mitosis, induce apoptosis, and produce regression in a fraction of cancers that arise at many sites including the ovary. Novel therapeutic targets that augment taxane effects are needed to improve clinical chemotherapy response in *CCNE1*-amplified high grade serous ovarian cancer (HGSOC) cells. In this study, we conducted an siRNA-based kinome screen to identify modulators of mitotic progression in *CCNE1*-amplified HGSOC cells that may influence clinical paclitaxel response. PLK1 is overexpressed in many types of cancer, which correlates with poor prognosis. Here, we identified a novel synthetic lethal interaction of the clinical PLK1 inhibitor BI6727 and the microtubule-targeting drug paclitaxel in HGSOC cell lines with *CCNE1*-amplification and elucidated the underlying molecular mechanisms of this synergism. BI6727 synergistically induces apoptosis together with paclitaxel in different cell lines including a patient-derived primary ovarian cancer culture. Moreover, the inhibition of PLK1 reduced the paclitaxel-induced neurotoxicity in a neurite outgrowth assay. Mechanistically, the combinatorial treatment with BI6727/paclitaxel triggers mitotic arrest, which initiates mitochondrial apoptosis by inactivation of anti-apoptotic BCL-2 family proteins, followed by significant loss of the mitochondrial membrane potential and activation of caspase-dependent effector pathways. This conclusion is supported by data showing that BI6727/paclitaxel-co-treatment stabilizes FBW7, a component of SCF-type ubiquitin ligases that bind and regulate key modulators of cell division and growth including MCL-1 and Cyclin E. This identification of a novel synthetic lethality of PLK1 inhibitors and a microtubule-stabilizing drug has important implications for developing PLK1 inhibitor-based combination treatments in *CCNE1*-amplified HGSOC cells.

## INTRODUCTION

High grade serous ovarian cancer (HGSOC) accounts for 70-80% of ovarian cancer-related deaths. Despite major improvements in the understanding of ovarian cancer carcinogenesis, most patients relapse after primary treatment and succumb to disease progression. In a recent study a total of 16,854 patients with HGSOC were analyzed. Median overall survival was 40.7 months among high grade patients [1]. Chemotherapy remains the principal form of adjuvant and neoadjuvant treatment for HGSOC. A significant advance in chemotherapeutic management was the introduction of paclitaxel to the treatment of HGSOC patients in which the combination of cisplatin and paclitaxel conferred a substantial survival advantage over cisplatin and cyclophosphamide. The toxicity profile favoring the carboplatin/paclitaxel regimen has now established this as standard of care in the first-line setting. Although changes to both chemotherapy schedules and routes of administration are associated with improved survival, it appears that a therapeutic ceiling with these drugs has been reached. Despite a good chemosensitivity of HGSOC, a significant frequency of these patients fails to respond to the primary treatment with taxanes. In recent years, inhibitors of poly(ADP-ribose) polymerase (PARP) have emerged as one of the most exciting new therapies for the treatment of HGSOC, based on the vulnerability of ovarian cancer cells to agents that interrupt DNA repair [2]. Bevacizumab, a humanized monoclonal antibody targeting vascular endothelial growth factor, showing improvement in progression-free survival in combination with standard chemotherapy has enriched the modern armament targeting HGSOC [3].

While mutations in oncogenes or tumor suppressor genes are relatively seldom in HGSOC except in genes coding for *TP53*, *BRCA1* and *BRCA2*, primary ovarian cancer tissues exhibit a high frequency of genomic structural variations, including multiple gains and losses of genomic DNA making this cancer type an example of chromosomal instability [4, 5]. Structural genomic variations are a frequent mechanism for the functional knockout of tumor suppressors in HGSOC, including *RB1* and *NF1* [6]. Mutations in *BRCA1* and *BRCA2* and, less frequent in other components of the homologous recombination (HR) pathway are the origin for defects in HR DNA repair pathway in approximately 50% HGSOC. The signaling pathways of the half of HGSOC that do not exhibit functional defects in HR remain to be elucidated. Approximately 30% of this subclass were found to have amplifications of the *CCNE1* gene, which codes for the G_1_/S-specific Cyclin E1, and this is likely an early event in the development of HGSOC [5].

Polo-like kinase 1 (PLK1) controls multiple phases of the cell-cycle progression [7, 8]. While the expression of PLK1 is below the limit of detection in normal tissues like heart, kidney, liver, brain, lung and pancreas, proliferating tissues like placenta, testis and bone marrow show high levels of PLK1 transcripts and protein [9, 10]. Early observations that linked PLK1 expression with cancer were from studies showing increased PLK1 expression in primary neoplastic tissues [9, 11]. PLK1 was shown to be overexpressed in a large spectrum of cancer types, including non-small cell lung cancer (NSCLC) [12], breast [13] ovarian [14] and head and neck squamous carcinomas [15] and melanoma [16]. Remarkably, high levels of PLK1 have been correlated with poor patient prognosis in different types of cancer including NSCLC [12], colon cancer [17] and hepatoblastoma [18]. Interestingly, a high risk of metastases has been associated with high PLK1 levels, implying a role for PLK1 in more aggressive tumors and the potential of PLK1 as a prognostic marker. The validation of PLK1 in multiple animal models revealed PLK1 as an important cancer target [19–22]. These observations prompted an intense search of the pharmaceutical industry for small molecule inhibitors of PLK1. The most advances PLK1 inhibitor in the clinic, Volasertib (BI6727) in combination with low dose cytarabine has received a Breakthrough Therapy designation from the FDA for its potential as a treatment for patients with untreated acute myeloid leukemia who are ineligible for intensive remission induction therapy [23]. In a study on normal ovaries (n=9), cystadenomas (n=17), borderline tumors (n=13) and ovarian carcinomas (n=77), the frequency of PLK1 expression was low in normal epithelium and borderline tumors, but in ovarian carcinomas 26% of the cancer tissues were PLK1-positive [14]. In ovarian cancer, a significant correlation between PLK1-positive cells and the histological grade was found [24]. The number of PLK1-positive cells was significantly higher in ovarian cancers designated as grade 3 than in cancers designated as grade 1 (P<0.001). Recently, in patients with ovarian cancer, comparable antitumor activity was observed between Volasertib treatment and the investigator's choice of single agent chemotherapy, including microtubule-targeting agents [25] suggesting that PLK1 could be considered as an attractive target for approaches aiming at the identification of synthetic lethality in the treatment of HGSOC.

The exploration of synergistic strategies that help to lower clinically relevant doses and to improve the response to taxane-based chemotherapy in HGSOC patients with amplification of *CCNE1* is a key aspect of our investigation. In this study, we performed an siRNA-based kinome screen in the OVCAR-3 cell line to identify regulators of mitotic progression and cell death that could augment the effect of taxanes such as paclitaxel.

## RESULTS

### A kinome-wide siRNA screen identifies modulators of cell growth and apoptosis in ovarian cancer cells with *CCNE1*-amplification

At first, we screened the Cyclin E expression of a small panel of ovarian cancer cells (Supplementary Figure 1) whose genotypes were analyzed recently [26]. For the subsequent analysis we selected an ovarian cancer cell line with *CCNE1*-amplification like OVCAR-3 cells exhibiting strong Cyclin E expression and a reasonable doubling time, which is an important prerequisite for a successful siRNA screening. We used a multi-step strategy to identify novel therapeutic targets in ovarian cancer cells that induce cell death and might improve taxane-based effects: (1) screening of 711 pools of siRNAs (Dharmacon kinome library) targeting individual human kinases; (2) specific hits were validated using single siRNAs (cherry picking) that made up the pool in the primary screening; and (3) assays for cell viability, cell death (Caspase-3/7 activity, Annexin staining) and cell cycle distribution (FACS measurements) of siRNA-treated OVCAR-3 cells (Supplementary Figure 2A). This cell line has high-level *CCNE1*-amplification and expression confirming previous data [27]. Our study was conducted to identify potent regulators of cell growth and apoptosis that were subsequently tested for their capability to synergize with paclitaxel.

In the primary screen of ovarian cancer cells with *CCNE1*-amplification, 48 h after transfection of the Dharmacon kinome library we first monitored cell viability and Caspase-3/7 activities of OVCAR-3 cells in a multiplexed assay (Supplementary Figure 2A-2C). As an additional test for apoptosis, 48 h after siRNA-transfection OVCAR-3 cells were stained for Annexin V (PE-Annexin V/7-AAD) and monitored by flow cytometry (Supplementary Figure 2A, 2D). For all three experiments, we determined the Top-20 list of targets whose depletion induced the most pronounced response on the growth and viability of cells (Supplementary Figure 2B-2D). We determined a high correlation between both apoptosis assays across triplicate plates (median correlation coefficient = 0.7) and a mean coefficient of repeatability for all genes tested of 0.1, indicating the high reproducibility of our screenings. Among the identified hits, the inhibition of Polo-like kinase 1 (PLK1), which is a key regulator of the progression through mitosis in mammalian cells [7, 28–29], was selected for further analysis based on its Top-20 ranking in all three assays with a particular focus on its highest potential to induce apoptosis in OVCAR-3 cells as determined by Annexin V staining (Supplementary Figure 2D).

Several studies have demonstrated that siRNAs are not always specific and can have off-target effects. To exclude off-target effects, we tested all 4 siRNA that were components of the pool used for the primary screen. All 4 siRNAs resulted in a pronounced downregulation of the protein level and a significant increase in Annexin V staining (Supplementary Figure 2E).

### Genomic profiling of paclitaxel-treated OVCAR-3 cells

We compared the microarray expression profiles of OVCAR-3 cells treated with low doses paclitaxel for 24 h with OVCAR-3 cells cultured in normal medium in order to identify kinase genes that are differentially expressed by using the Illumina HumanHT-12 v4 Expression BeadChip and to find out whether PLK1 might be a realistic target in paclitaxel-treated OVCAR-3 cells (Supplementary Figure 3A). To identify protein kinases that could act in concert with paclitaxel for the induction of synthetic lethality, we used for the microarray expression profiling sub-lethal doses of paclitaxel. At a concentration of 3.5 nM, the analysis of the microarray expression profile revealed an upregulation of classical regulators of G_2_/M like Aurora A, PLK4 and PLK1 compared to the DMSO control (Supplementary Figure 3A, Supplementary Table 1). Additional cell cycle regulators were also altered, including up-regulated CDK6, which has been previously described based on its aberrant expression and association with paclitaxel-based therapy in ovarian cancer patients [30, 31] and also including CDK7, which regulates the assembly of the CDK1/Cyclin B1 complex [32, 33]. These transcriptional alterations reflect the upregulation of mitotic regulators including PLK1 and stress response genes upon treatment of OVCAR-3 cells with low doses of paclitaxel. To analyze in more detail whether PLK1 could be an attractive target for a combinatorial approach with paclitaxel, we treated OVCAR-3 cells with increasing concentrations of paclitaxel for 24 h and observed escalating levels of PLK1 expression (10 nM: 140%) (Supplementary Figure 3B, upper panel) associated with an enrichment of cells in G_2_/M (Supplementary Figure 3B, lower panel).

Various lines of evidence suggest that cells held in mitosis for a prolonged period, for instance by microtubule targeting agents like paclitaxel, can undergo different fates including death by apoptosis [34]. Prolonged mitotic arrest is a key determinant of cell death with or without taxane treatment [35–37]. The treatment of OVCAR-3 cells with paclitaxel can not only prolong the duration of mitosis and thereby increase the percentage of apoptotic cells, it also increases the expression of PLK1 (Supplementary Figure 3A, 3B, upper panel), which is an attractive target in OVCAR-3 cells based on our kinome-wide siRNA screen (Supplementary Figure 2). Considering both aspects, we tested the combinatorial inhibition of PLK1 and the microtubule dynamics by paclitaxel in ovarian cancer cells with *CCNE1*-amplification.

### Small molecule inhibitors of PLK1 decrease the viability of ovarian cancer cells with *CCNE1*-amplification and sensitize cells to paclitaxel

**Figure 1:**
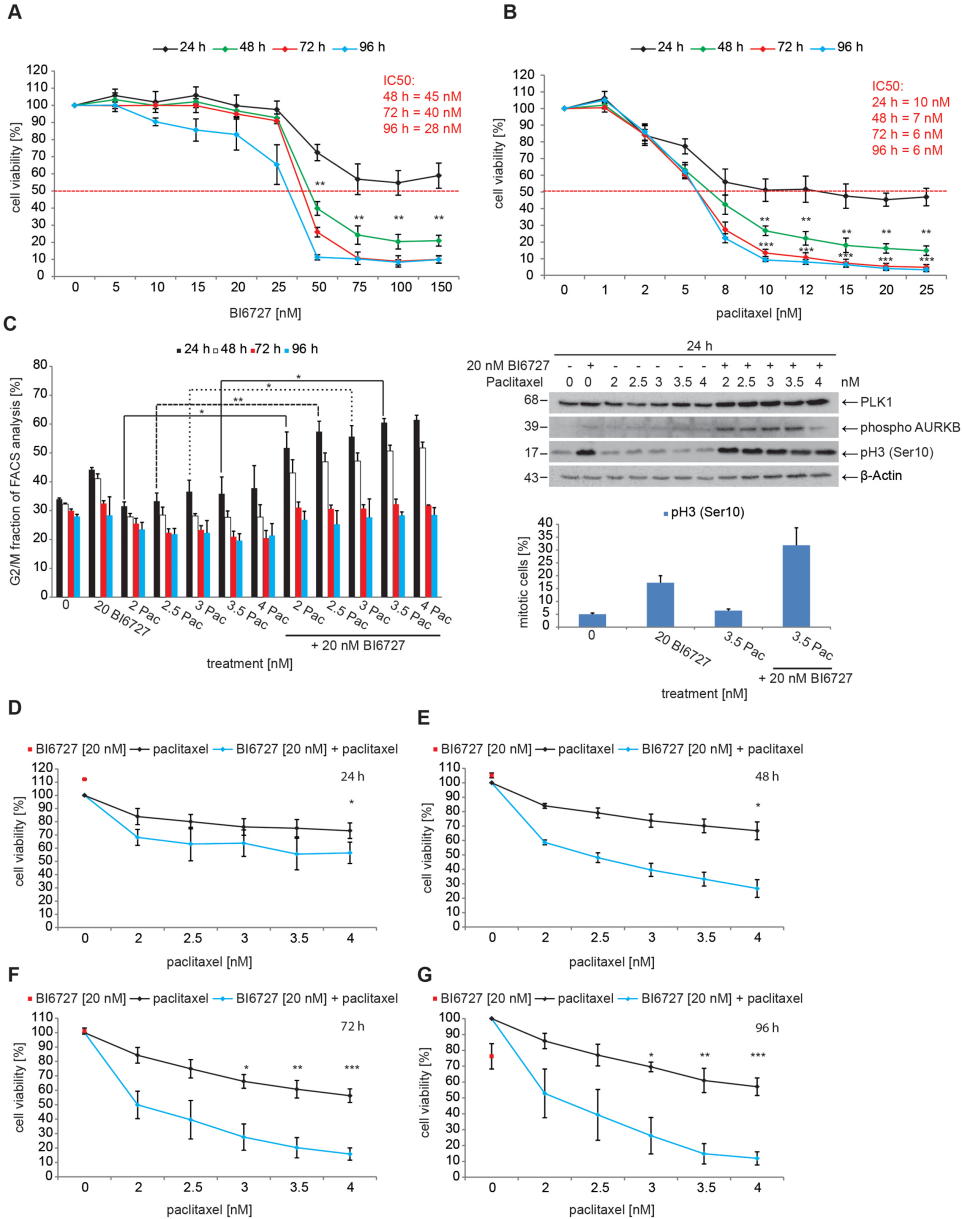
BI6727 treatment sensitizes ovarian cancer cells to paclitaxel **(A)** OVCAR-3 cells were treated with increasing concentrations of BI6727 or **(B)** of paclitaxel (Pac). Cell viability was measured over 4 d using the Cell Titer-Blue^®^ Cell Viability Assay. **(C)** (Left panel) The G_2_/M fraction was determined over 4 d post-treatment using flow cytometry. Measurements were statistically significant by two-tailed Student's *t*-test (^*^P ≤ 0.05; ^**^P ≤ 0.01). Each bar graph represents the mean value ± SEM (n=3). (Upper right panel) Endogenous levels of PLK1, Cyclin B1, phospho-Histone H3 and phospho-Aurora B were determined by immunoblotting. β-Actin served as loading control. (Lower right panel) The mitotic index was determined by measuring pH3(Ser10) levels. **(D-G)** OVCAR-3 cells were treated with either 20 nM BI6727 or increasing paclitaxel concentrations or both for 4 d. The cell viability was determined using the Cell Titer-Blue^®^ Cell Viability Assay. Measurements were statistically significant by two-tailed Student's *t*-test (^*^P ≤ 0.05; ^**^P ≤ 0.01; ^***^P ≤ 0.001). Each measurement represents the mean value ± SEM (n=3).

Although the inhibition of PLK1 functions by small-molecule inhibitors of its catalytic activity/subcellular localization or by RNAi-mediated depletion revealed that *differential* levels of PLK1 are required for the viability of cancer vs. normal cells [10, 38–42], the effect of PLK1 inhibition in HGSOC cells with *CCNE1*-amplification remains elusive. To study this aspect, at first the viability of OVCAR-3 cells following the treatment with the potent PLK inhibitor BI6727 [39] for 24 h, 48 h, and 72 h was determined. While BI6727 at low concentrations (≤10 nM) induced only a subtle effect in OVCAR-3 cells after 72 h of incubation, cell viability was significantly reduced to 26% at a dose of 50 nM (*P* < 0.01) and to 11% at 75 nM compared with DMSO-treated cells (*P* < 0.01; Figure 1A). After 96 h, more pronounced effects were observed: 65% at 25 nM, 11% at 50 nM and 10% at 75 nM (*P* < 0.01). While the treatment with paclitaxel for 72 h at concentrations ≤ 2 nM had only a small effect on the viability of OVCAR-3 cells, the treatment with 5 nM paclitaxel induced a significant reduction to 60% (*P* < 0.001) and to 13% at 10 nM compared with DMSO-treated cells (*P* < 0.001; Figure 1B). To evaluate whether these effects are cell-type specific, we treated a second cell line with *CCNE1*-amplification, COV318 cells, with increasing BI6727 concentrations. Due to the low proliferative activity of COV318 cells, we extended the observation period for our assays. Whereas BI6727 at low concentrations (≤10 nM) induced only a small effect in COV318 cells after 96 h of incubation, cell viability was significantly reduced to 48% at a dose of 50 nM (*P* < 0.001) and to 50% at 75 nM compared with DMSO-treated cells (*P* < 0.01; Supplementary Figure 4A). After 168 h more intense effects were measured: 47% at 25 nM, 37% at 50 nM and to 35% at 75 nM (*P* < 0.01). The treatment with 30 nM paclitaxel alone for 144 h reduced cellular viability to 24% (*P* < 0.05) and for 168 h to 18% (*P* < 0.01) compared with control cells (Supplementary Figure 4B).

The cell cycle analysis by FACS revealed that a treatment with 20 nM BI6727 for 24 h increases the fraction of OVCAR-3 cells in the G_2_/M phase from 34% to 44% (*P* < 0.01) (Figure 1C, left panel) due to prolonged mitotic arrest as evidenced by microscopical inspection. The treatment with increasing concentrations of paclitaxel (2-4 nM) led to a minor increase of OVCAR-3 cells in the G_2_/M phase from 32% to 38%. To further evaluate whether the cells are arrested in mitosis, we used phospho-histone H3 (pH3) as mitotic marker. The assessment by western blotting and FACS-based measurements revealed that cells that were treated with either agent alone showed only an increase of the pH3 signal for 20 nM BI6727 (Figure 1C, upper and lower right panels). The levels of mitotic markers like Cyclin B1, PLK1 and phospho-Aurora B remained nearly unchanged upon single agent-treatment. In contrast to the single treatments, the combination of 20 nM BI6727 with 2-4 nM paclitaxel induced a visible increase in the levels of the mitotic markers Cyclin B1, PLK1 and phospho-Aurora B (Figure 1C, upper right panel). Remarkably, the combinatorial treatment (24 h) of 3.5 nM paclitaxel and 20 nM BI6727 induced a strong increase to 61% of OVCAR-3 cells in the G_2_/M phase (Figure 1C, left panel, *P* < 0.01) and to 33% by FACS-based determination of pH3-positive cells (Figure 1C, lower right panel).

To follow up upon this pronounced combinatorial effect, we tested the viability of HGSOC cells following single or combinatorial treatments. We analyzed the effect of 20 nM BI6727 together with paclitaxel in concentrations from 2-4 nM on the viability of OVCAR-3 cells for 24-96 h (Figure 1D-1G). BI6727 alone at 20 nM affected the viability of OVCAR-3 cells compared with controls (24 h, 112%, 48 h, 105%, 72h, 101%; 96 h, 76%) (Figure 1D-1G). For comparison, after a treatment for 96 h paclitaxel alone at concentrations between 2 and 4 nM reduced cell viability to 86% (2 nM) or 57% (4 nM) (*P* < 0.05) (Figure 1G). Interestingly, together with 20 nM BI6727, 2 nM paclitaxel reduced cell proliferation to 50% (*P* < 0.05), with 2.5 nM to 40% (*P* < 0.05), with 3 nM to 28% (*P* < 0.05), with 3.5 nM to 20% (*P* < 0.01) and with 4 nM to 16% (*P* < 0.001) (Figure 1F). The combination of 4 nM paclitaxel and 20 nM BI6727 for 96 h revealed a very pronounced effect with a residual viability of 12% (*P* < 0.001) (Figure 1G). The treatment of OVCAR-3 cells with BI6727 decreased the dose of paclitaxel required to reduce cancer cell growth by 50% (GI50) by 2.8–3.0-fold. Therefore, the application of the PLK1 inhibitor BI6727 sensitized ovarian cancer cells to paclitaxel in a synergistic manner (combination index <1).

Moreover, COV318 cells were subjected to a combinatorial treatment with 20 nM BI6727. Interestingly, together with 20 nM BI6727, 1 nM paclitaxel reduced cell proliferation after 120 h to 67% (compared to 97%, 1 nM paclitaxel alone), with 2 nM to 52% (*P* < 0.05) (compared to 93%, 2 nM paclitaxel alone), and with 5 nM to 47% (*P* < 0.01) (compared to 80%, 5 nM paclitaxel alone), (Supplementary Figure 4C). After 144 h the combinatorial incubation with 20 nM BI6727 and 5 nM paclitaxel reduced cell proliferation to 43% (*P* < 0.05) (Supplementary Figure 4D). The treatment of COV318 cells with BI6727 decreased the dose of paclitaxel required to reduce cancer cell growth by 50% (GI50) from 25 nM to 2 nM. A pronounced inhibition of cell viability using paclitaxel was achieved in combination with BI6727 indicating a synergistic action (combination index <1). Importantly, these observations were not cell-type specific. Together, this set of experiments reveals a novel cooperation of PLK1 inhibition and microtubule-targeting drugs like paclitaxel to reduce cell viability in ovarian cancer cell lines with *CCNE1*-amplification.

### Paclitaxel and a PLK1 inhibitor cooperate to induce apoptosis in ovarian cancer cell lines with *CCNE1*-amplification

**Figure 2:**
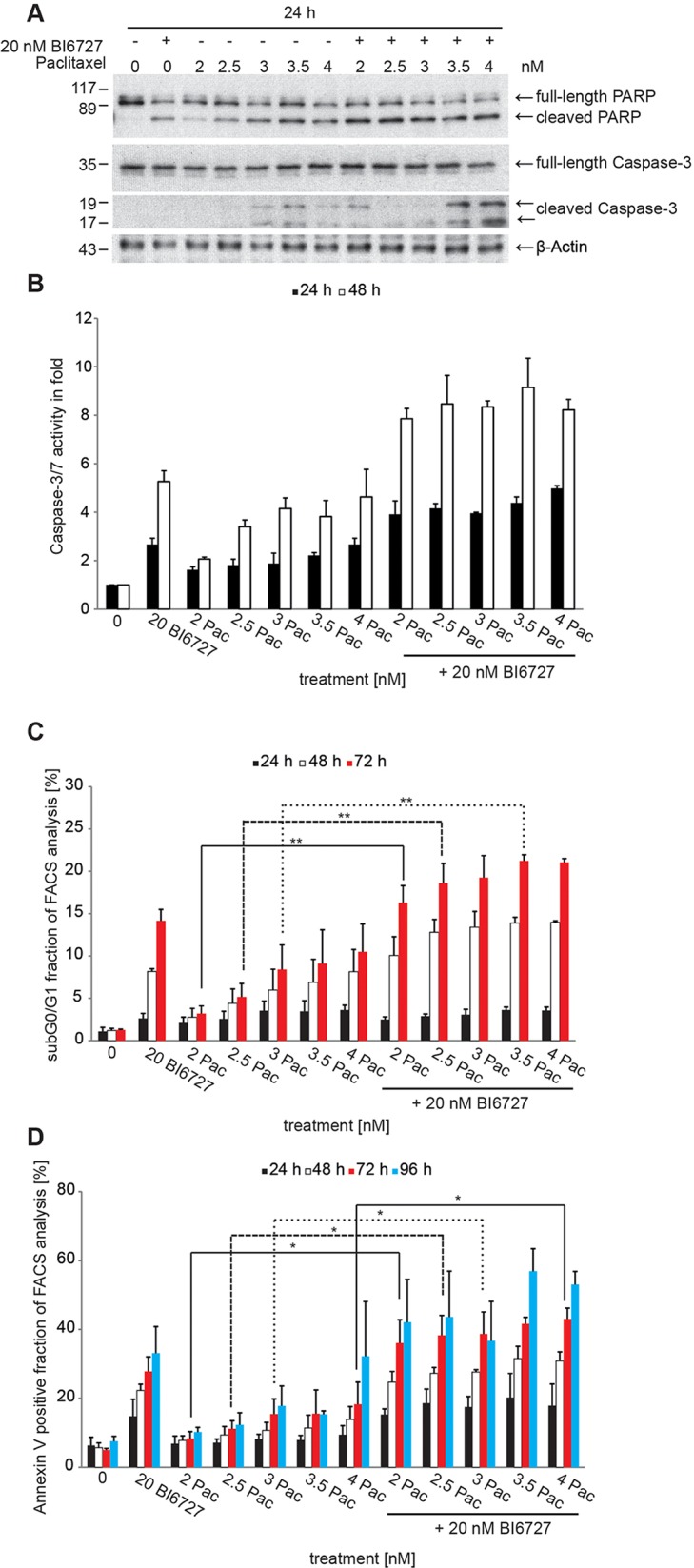
The combinatorial treatment of BI6727 and paclitaxel induces pronounced levels of apoptosis **(A)** OVCAR-3 cells were treated with 20 nM BI6727, increasing paclitaxel concentrations or both. Apoptosis was analyzed 24 h post-treatment by western blotting for Caspase-3, full-length and cleaved PARP. β-Actin served as loading control. **(B)** Caspase-3/7-activity in whole cell lysates of 20 nM BI6727, paclitaxel or both treated OVCAR-3 cells was measured 24 h and 48 h post-treatment using the Caspase-Glo^®^ 3/7 Assay. Each bar graph represents the mean value ± SEM (n=2). Apoptosis was validated **(C)** by measuring the sub G_0_/G_1_ fractions or **(D)** by PE Annexin V staining. Measurements were statistically significant by two-tailed Student's t-test (^*^P ≤ 0.05; ^**^P ≤ 0.01). Each bar graph represents the mean value ± SEM (n=3).

To examine the apoptotic response of cells arrested in the G_2_/M phase induced by a combinatorial treatment of paclitaxel and a PLK1 inhibitor, at first we treated OVCAR-3 cells with increasing doses of paclitaxel (2-4 nM) or 20 nM BI6727 and analyzed the induction of apoptosis by monitoring Caspase-3 cleavage as well as its downstream target, cleaved PARP, in western blot experiments (Figure 2A). While the cleavage of full-length Caspase-3 and PARP, respectively, showed low to moderate levels at paclitaxel concentrations between 2 and 4 nM for 24 h, the combinatorial treatment with 20 nM BI6727 induced strong cleavage of PARP and Caspase-3 in particular at concentrations of 3.5 and 4 nM (Figure 2A).

Moderate Caspase-3/7 activation could be detected after treatment of cells with paclitaxel alone (Figure 2B; 2 nM activation to 1.6-fold, 2.5 nM activation to 1.8-fold, 3 nM activation to 1.9-fold, 3.5 nM activation to 2.2-fold, 4 nM activation to 2.7-fold). This activation could be enhanced by co-incubation with 20 nM BI6727 (20 nM BI6727 + 2 nM paclitaxel activation to 3.9-fold compared with 2 nM paclitaxel, *P* = 0.1, 20 nM BI6727 + 2.5 nM paclitaxel to 4.2-fold compared with 2.5 nM paclitaxel, *P* = 0.1, 20 nM BI6727 + 3.0 nM paclitaxel to 4-fold compared with 3 nM paclitaxel, *P* = 0.1, 20 nM BI6727 + 4 nM paclitaxel to 5-fold compared with 4 nM paclitaxel, *P* = 0.1).

In addition, the cell cycle analysis by FACS revealed also a significant increase of the sub G_0_/G_1_ peak representing the apoptotic cell population comparing paclitaxel versus paclitaxel/BI6727-treated cells (Figure 2C). We extended our evaluation to a genetic approach for PLK1 inhibition. The silencing of PLK1 was performed by using the most efficient PLK1-specific siRNA sequence obtained from “cherry picking” of the Dharmacon pool (Supplementary Figure 5A). PLK1 depletion by RNAi significantly increased paclitaxel-triggered apoptosis as documented by a significant increase of the sub G_0_/G_1_ fraction (Supplementary Figure 5B, 5C).

Furthermore, cells were stained with PE Annexin V/7-AAD and analyzed using a flow cytometer (FACS). After incubation of OVCAR-3 cells with paclitaxel at concentrations between 2 and 4 nM for different time intervals we observed a small increase of Annexin V-positive cells: 24 h (2 nM paclitaxel 7%, 2.5 nM 7%, 3 nM 8%, 3.5 nM 8%, 4 nM 9%), 48 h (2 nM paclitaxel 8%; 2.5 nM 9%, 3 nM 11%; 3.5 nM 11%; 4 nM 14%) 72 h (2 nM paclitaxel 8%; 2.5 nM 11%, 3 nM 15%; 3.5 nM 16%; 4 nM 18%) and 96 h (2 nM paclitaxel 10%, 2.5 nM 12%, 3 nM 18%, 3.5 nM 15%, 4 nM 32%), suggesting that up to 30% of the paclitaxel-treated cells had entered the apoptotic pathway versus 8% of apoptosis in control cells (Figure 2D). The co-incubation of cells with 20 nM BI6727 and paclitaxel induced a more pronounced apoptosis up to 53% at a concentration of 4 nM paclitaxel: 24 h (2 nM paclitaxel 15%, *P* < 0.01; 2.5 nM 19%, 3 nM 18%; 3.5 nM 20%; 4 nM 18%), 48 h (2 nM paclitaxel 25%; 2.5 nM 27%, 3 nM 28%; 3.5 nM 32%; 4 nM 31%) 72 h (2 nM paclitaxel 36%; 2.5 nM 38%, 3 nM 39%; 3.5 nM 42%; 4 nM 43%) and 96 h (2 nM paclitaxel 42%; 2.5 nM 44%, 3 nM 37%; 3.5 nM 57%; 4 nM 53%). Together, this set of experiments shows that PLK1 inhibition and paclitaxel cooperate in a synergistic manner to induce apoptosis in ovarian cancer cell lines with *CCNE1*-amplification based on our Annexin V experiments (combination index <1).

### Combinatorial treatment with paclitaxel and the PLK1 inhibitor BI6727 induce mitochondrial apoptosis

**Figure 3:**
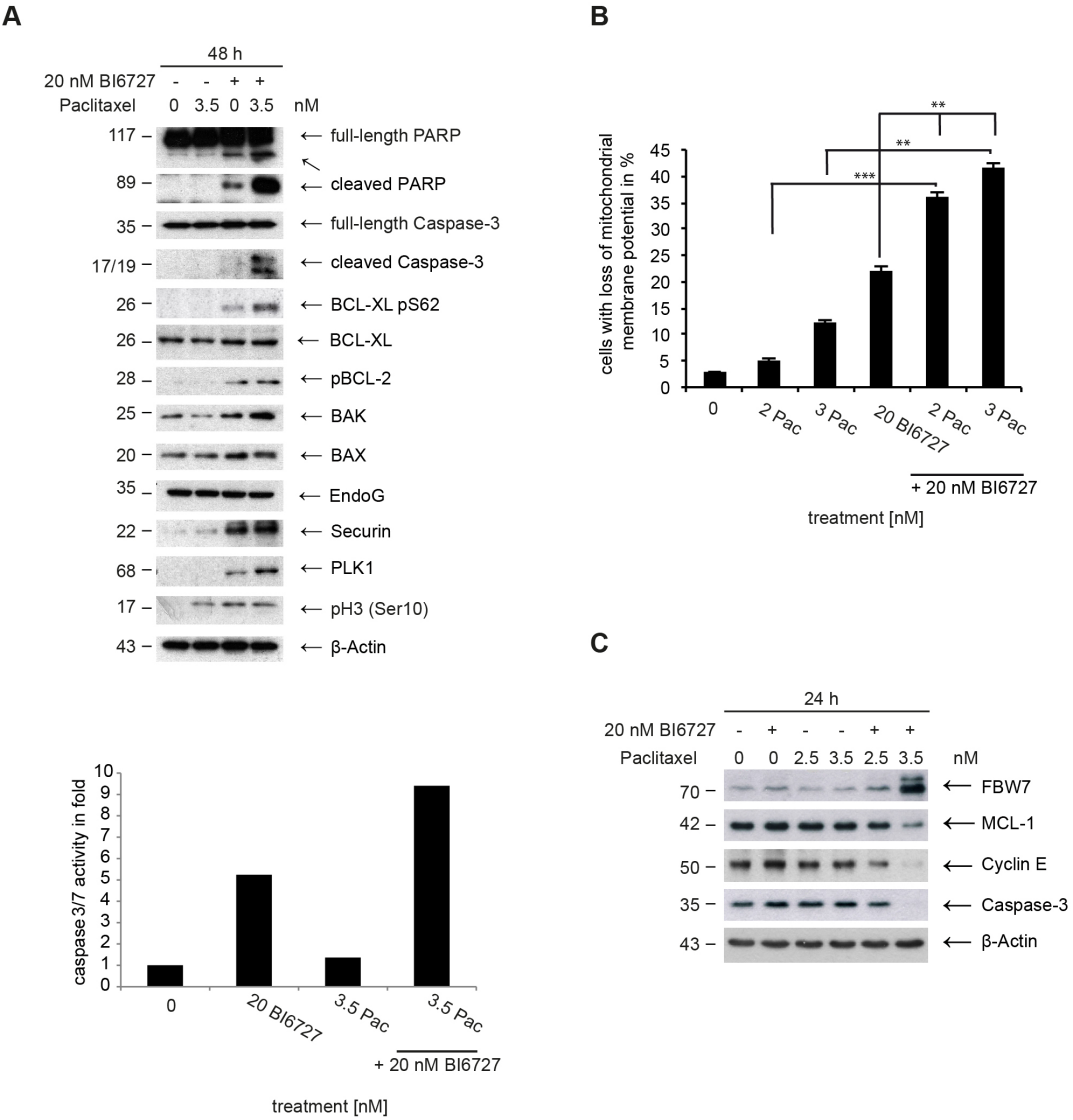
Analysis of apoptotic signaling in ovarian cancer cells **(A)** (upper panel) Whole cell lysates of OVCAR-3 cells treated with BI6727 or 3.5 nM paclitaxel or both were analyzed evaluating marker proteins for mitochondrial-mediated apoptosis. Endogenous levels of PARP, cleaved PARP, Caspase-3, cleaved-Caspase-3, BCL-X_L_ pS62, BCL-X_L_, pBCL-2, BAK, BAX, EndoG, Securin, PLK1, pHistone H3 and ß-Actin were determined by immunoblotting. Caspase-3/7-activity in whole cell lysates of 20 nM BI6727, paclitaxel or both treated OVCAR-3 cells was measured 48 h post-treatment using the Caspase-Glo^®^ 3/7 Assay. **(B)** Evaluation of the mitochondrial membrane potential. The data are presented as means ± SD. Statistical analysis among treatment groups was performed. ^**^ (*P*< 0.01), ^***^ (*P*< 0.001). **(C)** Endogenous levels of FBW7, MCL-1, Cyclin E, Caspase-3 and ß-Actin were determined by immunoblotting using specific antibodies.

Mitochondria play essential roles for the induction of apoptosis in mammalian cells. Members of the B-cell lymphoma 2 (BCL-2) family trigger the release of proteins from the space between the mitochondrial inner and outer membrane [43]. This process activates caspases that dismantle cells and signal efficient phagocytosis of cell corpses. The activation of either BCL-2- associated X protein (BAX) or BCL-2 antagonist or killer (BAK) is a key event for mitochondrial outer membrane permeabilization (MOMP) as cells lacking both proteins fail to undergo MOMP and apoptosis in response to apoptotic stimuli. First, we examined members of the anti-apoptotic BCL-2 family, which contribute to the regulation of the mitotic arrest [44]. Indeed, while 3.5 nM paclitaxel had no impact on the phosphorylation of BCL-X_L_ and of BCL-2, 20 nM BI6727 induced low levels of phosphorylation and the combinatorial treatment prompted elevated levels of both phosphorylation events (Figure 3A, upper panels) associated with strong Caspase-3/7 activity (Figure 3A, lower panel). The mitochondrial membrane potential (ΔΨ_m_) is an important indicator of mitochondrial function and dysfunction. Using a flow cytometry method for the quantitative measurement of ΔΨ_m_ which utilizes the lipophilic, cationic, fluorescent probe 3,3′-dihexyloxacarbocyanine iodide (DiOC_6_(3)) we observed a significant loss of ΔΨ_m_ in the combinatorial treatment compared to the use of single agents (Figure 3B)

New studies have identified a pathway involving the protein Myeloid cell leukemia 1 (MCL-1), an anti-apoptotic member of the BCL-2 family of apoptosis-regulating proteins, that couples the timing of mitosis to the induction of apoptosis [44]. Pro-survival members, including MCL-1, inhibit apoptosis by blocking the cell death mediators BAX and BAK. When uninhibited, BAX and BAK permeabilize the outer mitochondrial membrane, which releases pro-apoptotic factors that activate caspases. During prolonged mitotic arrest, MCL-1 protein levels decline substantially, through polyubiquitination mediated by the tumor-suppressor protein FBW7, stimulating cell death. MCL-1 levels were not reduced in BI6727- or paclitaxel-treated cells (Figure 3C). PLK1 was recently shown to regulate the stability of FBW7 leading to the modulation of N-Myc levels [45]. We then sought to examine how PLK1 might affect FBW7 levels in ovarian cancer cells. Inhibition of PLK1 by BI6727 increased the protein abundance of FBW7 (Figure 3C). The addition of the 26S proteasome inhibitor, MG132, almost rescued the loss of MCL-1 caused by PLK1 inhibition, supporting the model that PLK1 regulates MCL-1 expression primarily via a posttranslational mechanism in OVCAR-3 cells (data not shown). The co-treatment including BI6727 and paclitaxel caused the most pronounced stabilization of FBW7 compared to single treatments and led to a pronounced downregulation of the MCL-1 level (Figure 3C). High levels of PLK1, Securin and phopho-H3 in co-treated cells suggest that the inactivation of anti-apoptotic BCL-2 family members (downregulation of MCL-1, phosphorylation of BCL-X_L_ and BCL-2) occurs in mitosis (Figure 3A). In summary, BI6727 and paclitaxel cooperate to activate the mitochondrial pathway leading to the activation of Caspase-3 (Figures 2A, 3A).

### Paclitaxel and PLK1 inhibition do not synergize to reduce cell viability in an ovarian cancer cell line without *CCNE1*-amplification

To study the effect of PLK1 inhibition and/or paclitaxel treatment on the viability of cells without

*CCNE1*-amplification, we tested Ovsaho cells (Supplementary Figure 1), which is a cell line showing the major genomic characteristics of HGSOC, and thus this cell line seems to be well suited as *in vitro* model for HGSOC [26]: This cell line has a TP53 mutation and its copy-number profile correlates well with the mean copy-number alterations of ovarian tumors. Its alteration pattern in the ovarian cancer-specific gene set matches TCGA samples. The IC_50_ was reached at concentrations >150 nM BI6727 in Ovsaho cells after 96 h of treatment (Supplementary Figure 6A). The incubation with >10 nM paclitaxel for 72 h and 96 h reduced cell viability by more than 50% (Supplementary Figure 6B). Remarkably, the combinatorial treatment (24-72 h) of 20-30 nM paclitaxel and 75 nM or 100 nM BI6727 did not induce a significant increase of Ovsaho cells in the G_2_/M phase (Supplementary Figure 6C). Furthermore, the combination of 75 nM or 100 nM BI6727 with increasing concentrations of paclitaxel (20-30 nM) for a period of 24 h, 48 h and 72 h did not reduce the cell viability compared to single treatments (Supplementary Figure 6D-6F).

To examine the apoptotic response of Ovsaho cells induced by a combinatorial treatment of paclitaxel and a PLK1 inhibitor, we treated cells with increasing doses of paclitaxel (10-30 nM) or 100 nM BI6727 and analyzed the induction of apoptosis by monitoring cleaved PARP in western blot experiments (Supplementary Figure 7A). While low levels of PARP cleavage were visible at paclitaxel concentrations between 10-30 nM at 72 h, the combinatorial treatment with 100 nM BI6727 induced a small increase of PARP cleavage (Supplementary Figure 7A). In line with these observations, the cell cycle analysis by FACS did not show a significant increase of the sub G_0_/G_1_ peak representing the apoptotic cell population comparing paclitaxel versus paclitaxel/BI6727-treated cells (Supplementary Figure 7B). Furthermore, cells were stained with PE Annexin V or PE Annexin V/7-AAD and analyzed using a flow cytometer (FACS) (Supplementary Figure 7C, 7D). Both measurements did not provide evidence for a significant increase of apoptosis under the combinatorial treatment compared to single treatments showing a remarkable difference to both ovarian cancer cell lines with *CCNE1*-amplification (OVCAR-3, COV318).

### The impact of combined treatment with PLK1 inhibitor and paclitaxel on the clonogenic survival of ovarian cancer cells

**Figure 4:**
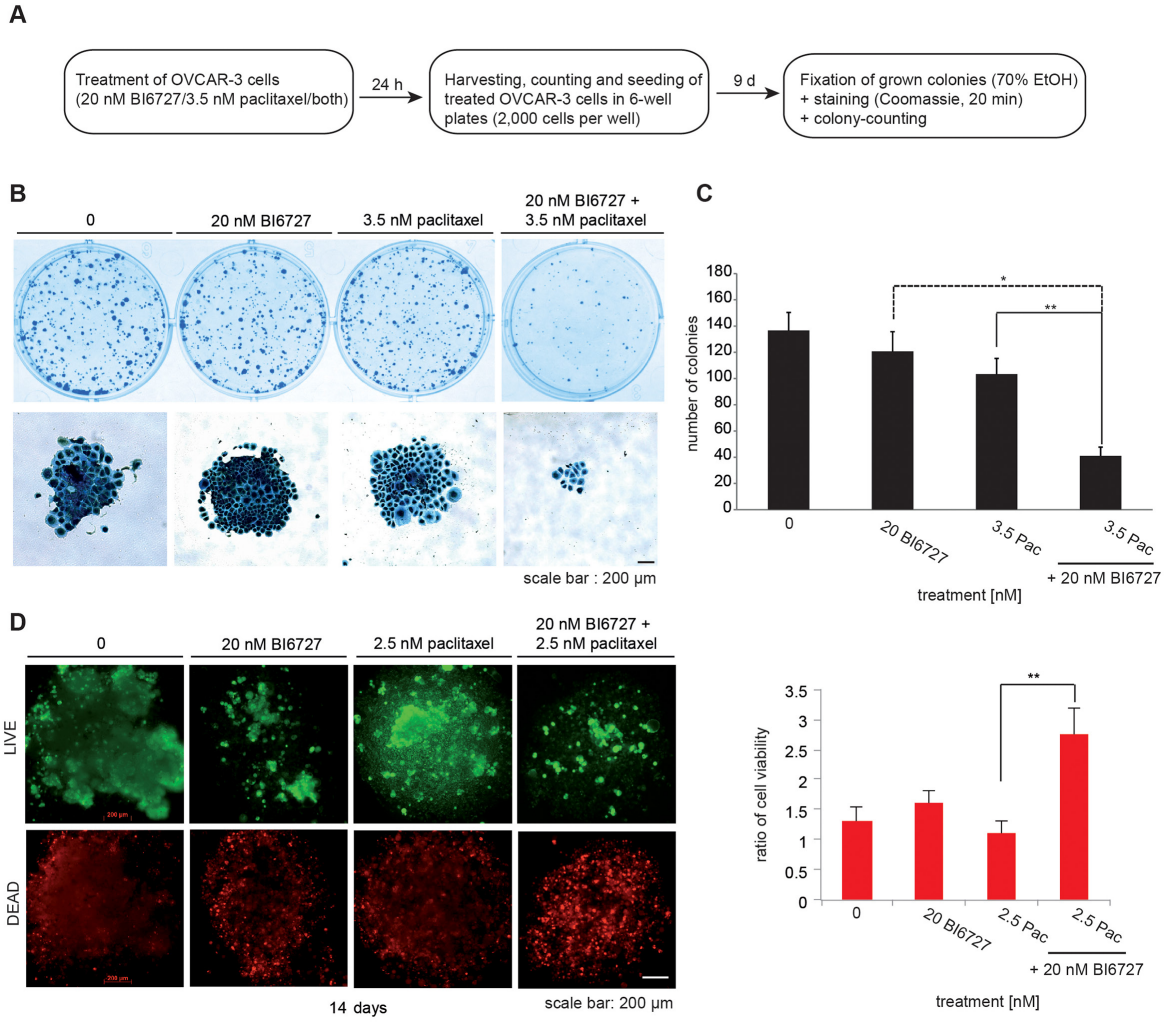
Combinatorial treatment of OVCAR-3 cells with 20 nM BI6727 and 3 5 nM paclitaxel displays long lasting inhibition of cell growth. **(A)** Experimental set up of long-term analyzes. **(B)** Coomassie stained regrown colonies of OVCAR-3 cells treated with 20 nM BI6727, 3.5 nM paclitaxel or both for 24 h. **(C)** The number of colonies was determined. Numbers were statistically significant by two-tailed Student's *t*-test (^*^P≤ 0.05; ^**^P≤ 0.01). Each bar graph represents the mean value ± SEM (n=3). **(D)** OVCAR-3 cells were grown as 3-D culture over 14 d and treated with 20 nM BI6727, 2.5 nM paclitaxel or both for 5 d. Cells were stained using the LIVE/DEAD viability/cytotoxicity kit and ratios of viable/dead cells were calculated. Measurements were statistically significant by two-tailed Student's t-test (^*^P≤ 0.05; ^**^P≤ 0.01). Each bar graph represents the mean value ± SEM (n=3).

To study the tumorigenic potential of cells treated with BI6727 and paclitaxel, we tested for the ability of cells to survive and to form colonies. Briefly, for this colony formation assay, cells were cultured for 24 h in the presence of 20 nM BI6727 or 3.5 nM paclitaxel or 20 nM BI6727/3.5 nM paclitaxel and then re-plated in 6 well-plates (2000 cells/well) in inhibitor-free medium (Figure 4A). After 9 d the resulting colonies were stained with Coomassie blue and phenotypically inspected using a phase-contrast microscope (Figure 4B). The results of the long-term experiments revealed that BI6727 and paclitaxel acted together to significantly reduce the colony forming ability (20 nM BI6727 121 colonies; 3.5 nM paclitaxel 103 colonies; 3.5 nM paclitaxel/20 nM BI6727 41 colonies, *P* < 0.01) (Figure 4C) and to synergistically reduce cell viability in a three-dimensional (3-D) multicellular model (20 nM BI6727 123%; 2.5 nM paclitaxel 84%; 2.5 nM paclitaxel/20 nM BI6727 212%, *P* < 0.01) (Figure 4D), which are widely used as avascular tumor model for metastasis and invasion research.

### PLK1 inhibitor and paclitaxel synergistically induce apoptosis and suppress clonogenic growth in primary, patient-derived ovarian cancer cell cultures

**Figure 5:**
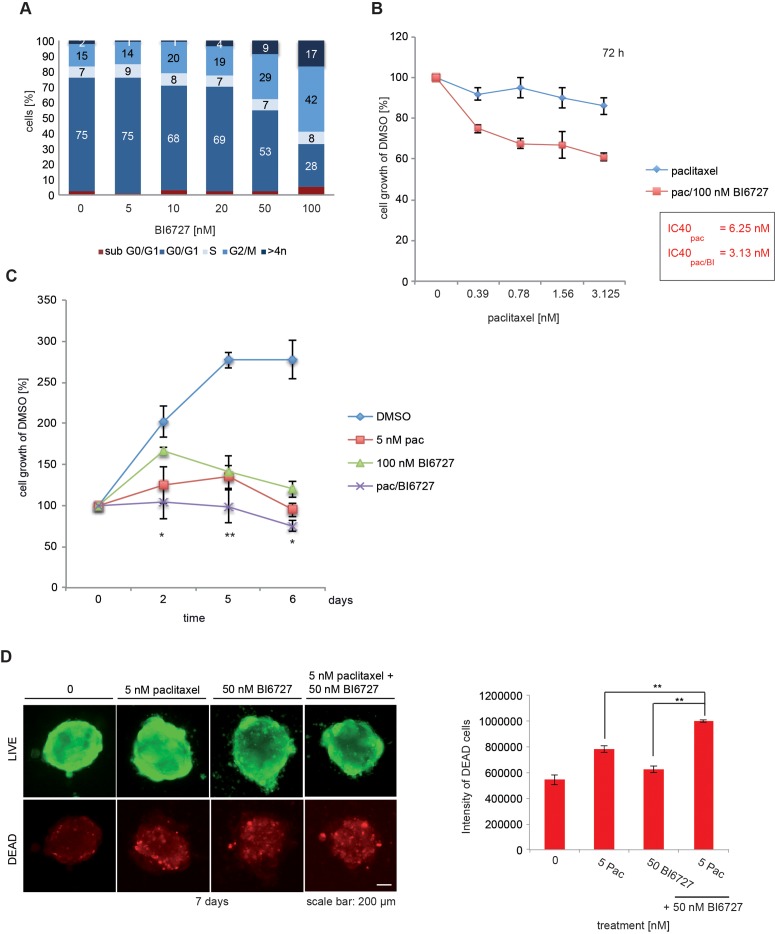
BI6727 treatment sensitizes patient-derived HGSOC cells to paclitaxel **(A)** Primary tumor cells were treated with increasing BI6727 concentrations and the cell cycle distribution was analyzed by flow cytometry. **(B)** Primary tumor cells were either treated with increasing paclitaxel concentrations or both (increasing concentrations of paclitaxel and 100 nM BI6727). The cell viability was determined for 72 h or **(C)** over a period of 6 d using the Cell Titer-Blue Cell^®^ Viability Assay. **(D)** 3-D cultures grown out of primary tumor cells were treated with either 5 nM paclitaxel, 50 nM BI6727 or both for 7 d. Cells were stained and fluorescence intensities of dead cells were determined. Measurements were statistically significant by two-tailed Student's *t*-test (^*^P≤ 0.05; ^**^P≤ 0.01). Each bar graph represents the mean value ± SEM (n=3).

To evaluate the physiological relevance of our observations, we extended our study to a primary ovarian cancer cell culture established from tumor specimen derived from patients diagnosed with HGSOC. The incubation with BI6727 at concentrations between 5 and 100 nM for 24 h increased the fraction of HGSOC cells in the G_2_/M phase from 14% to 42% (Figure 5A). Notably, paclitaxel and BI6727 synergized to reduce cell growth in short-term (72 h) (3.125 nM paclitaxel 86%; 3.125 nM paclitaxel/100 nM BI6727 61%) and long-term (5 nM paclitaxel 95%; 100 nM BI6727 120%; 5 nM paclitaxel/100 nM BI6727 75%, *P* < 0.05) experiments (6 days) (Figure 5B, 5C). The analysis of the 3-D cultures revealed a significant increase of dead cells by the combinatorial treatment compared to single agents (Figure 5D). The co-treatment with paclitaxel and BI6727 reduced both cell viability and 3-D colony formation in the primary ovarian culture (Figure 5B-5D). Furthermore, we analyzed a sample derived from a second patient diagnosed with HGSOC (Supplementary Figure 8). The co-treatment with paclitaxel and BI6727 confirmed the significant reduction of cell viability and 3-D colony formation (Supplementary Figure 8A-8C) supporting the clinical relevance of this combinatorial approach in the primary ovarian culture.

### The inhibition of PLK1 supports a reduction of the paclitaxel-induced neurotoxicity

**Figure 6:**
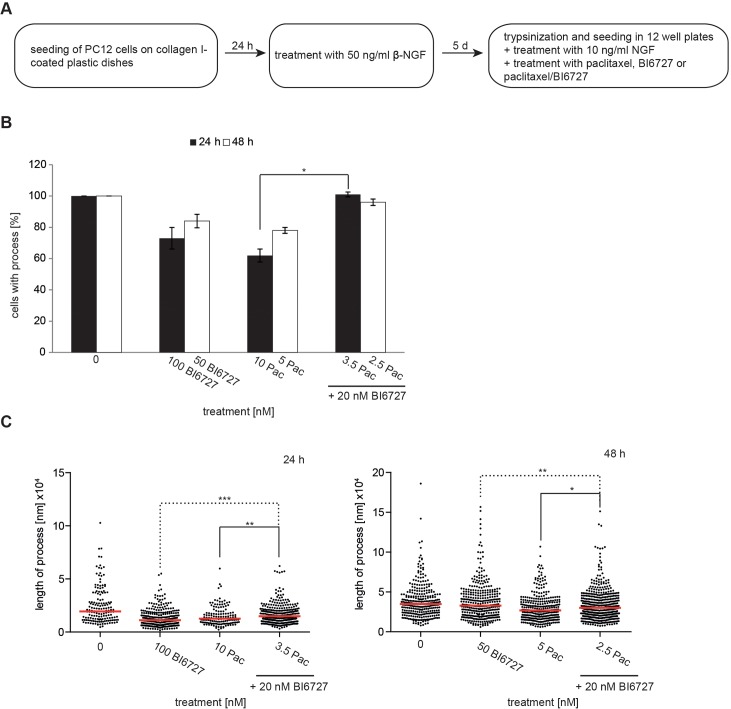
The combinatorial treatment of paclitaxel and BI6727 reduces neurotoxicity **(A)** Experimental set up of neurotoxicity analysis using PC-12 cells. **(B)** PC-12 cells were differentiated into neurons using β-NGF. The outgrowth of neurites was analyzed 24 h and 48 h post-treatment and cells displaying elongations were counted. Numbers were statistically significant by two-tailed Student's t-test (^*^P≤ 0.05). Each bar graph represents the mean value ± SEM (n=3). **(C)** The lengths of neurites were measured 24 h/48 h post-treatment and depicted as Scatter plots. Measurements were statistically significant by Student's *t*-test (^*^P≤ 0.05; ^**^P≤ 0.01; ^***^P≤ 0.001).

As paclitaxel is known to harm neuronal cells in patients, we investigated whether a combinatorial treatment with paclitaxel and BI6727 might reduce neuronal toxicity. To address this aspect, we used the Nerve Growth Factor (NGF)-induced neurite outgrowth assay in the rat pheochromocytoma cell line PC-12, which is an established *in vitro* model and is often used to study neuronal differentiation and neurotoxicity [46] (Figure 6A). A significant reduction of the number of cells expressing neurites was determined with 5 nM and 10 nM paclitaxel to 78% and 62%, respectively (*P*<0.05). Subsequently, we compared the neurotoxic effect of different drugs at their IC_50_ in OVCAR-3 cells: 10 nM paclitaxel vs. 3.5 nM paclitaxel and 20 nM BI6727. The co-incubation of 3.5 nM paclitaxel and 20 nM BI6727 showed a positive effect, increasing the percentage of neurite-forming cells from 62% (10 nM paclitaxel) to 101% (3.5 nM paclitaxel/ 20 nM BI6727) (*P*<0.05) (Figure 6B). Furthermore, we also observed a positive effect on the length of processes 12.63 nm vs. 14.72 nm comparing 10 nM paclitaxel vs. 3.5 nM paclitaxel/20 nM BI6727 (Figure 6C). Thus, the reduction of the paclitaxel concentration made possible by the addition of BI6727 reduces the neurotoxicity in the PC-12-based model system.

## DISCUSSION

Paclitaxel is a front-line drug for ovarian cancer chemotherapy, along with the platinum agents. Defining the appropriate dosage schedule for paclitaxel in chemo-naïve and recurrent ovarian cancer and elucidating the mechanisms of taxane resistance are areas of intense research. Importantly, avoiding troublesome / irreversible adverse effects and improving clinical efficacy are of utmost interest for the therapy of HGSOC. Peripheral neurotoxicity is the major non-hematological adverse effect of taxane, which is often painful and sometimes irreversible. In view of unwanted side-effects associated with conventional chemotherapy, decreasing the dose of paclitaxel is a valuable strategy to limit neurotoxicity and to improve the clinical efficacy in combinatorial treatments. In this study, we utilized a human siRNA library targeting 711 kinases for the screening of ovarian cancer cells with *CCNE1*-amplification as a model to identify protein kinase targets suitable for new synthetic lethal drug interactions that could optimize the paclitaxel-mediated therapeutic effects. We identified a novel combinatorial approach of paclitaxel and the small molecule inhibitor of PLK1, BI6727, to induce growth inhibition and apoptosis in ovarian cancer cell lines with *CCNE1*-amplification. The calculation of combination indexes suggests synergistic lethality, which indicates the potency of this drug combination. Importantly, the examination of primary ovarian cancer cells revealed cooperative anti-tumor cell activity of BI6727 and paclitaxel causing synergistic apoptosis and emphasizing the clinical relevance of our investigation. In contrast, the combinatorial treatment of an ovarian cancer cell line without *CCNE1*-amplification with paclitaxel and BI6727 did not provide evidence for synthetic lethality.

Mitochondrial outer membrane permeabilization (MOMP) is often essential for the activation of the caspase proteases that are responsible for apoptotic cell death. Various intermembrane space (IMS) proteins are released. Following MOMP by pro-apoptotic BAX or BAK, additional regulatory mechanisms govern the mitochondrial release of IMS proteins and caspase activity. MOMP typically leads to cell death irrespective of caspase activity by causing a progressive decline in mitochondrial function. In our study, we have tried to further our knowledge on the mechanisms that contribute to an induction of apoptosis upon BI6727/paclitaxel treatment, which induces a pronounced mitotic arrest and cell death. BI6727 and paclitaxel cooperate to disable anti-apoptotic BCL-2 family proteins by inactivating BCL-2 and BCL-X_L_ via phosphorylation and by downregulating MCL-1 shifting the balance between pro- and anti-apoptotic BCL-2 family proteins toward apoptosis. In concordance with our data, it has been shown that upon mitotic arrest the phosphorylation and subsequent ubiquitination of MCL-1 lead to its degradation [37, 44, 47]. Moreover, it could be demonstrated that phospho-defective BCL-2 and BCL-X_L_ mutants block mitotic cell death compared to the corresponding wild-type proteins and the phosphorylation of BCL-X_L_ reduces its capacity to antagonize pro-apoptotic BAX [48–50].

Given our current knowledge on the regulation of apoptosis during prolonged mitotic arrest induced by microtubule-targeting drugs two strategies to increase the efficacy of spindle-poisons like paclitaxel are 1) extending the duration of the mitotic arrest and 2) accelerating the degradation of the apoptotic timer. In other words, in order to shift the balance from cell survival to apoptosis, the rate of Cyclin B1 degradation needs to be slowed and the rate of MCL-1 degradation must be increased. Various signaling pathways are involved in the regulation of MCL-1 stability: When MCL-1 expression is increased following mitotic arrest, its degradation is controlled by different regulators [44]. FBW7, the substrate-binding component of a ubiquitin ligase complex, targets MCL-1 for degradation by the 26S proteasome, whereas the deubiquitinase USP9X reverses the polyubiquitinating activity of the FBW7 complex [51]. During mitotic arrest, the activities of Jun N-terminal kinase (JNK), the mitogen-activated protein kinase (MAPK) family member p38, and casein kinase II (CKII) are upregulated, increasing the phosphorylation of MCL-1, interaction with FBW7, and the subsequent polyubiquitination of MCL-1, which leads to degradation via the proteasome and continually decreasing concentrations of MCL-1 during mitotic arrest [52]. A recent study revealed that PLK1 reduces FBW7 stability through promotion of its phosphorylation and autopolyubiquitination [45]. To evaluate the role of PLK1 for the stability of FBW7 in ovarian cancer cells, we tested BI6727 for its capacity to trigger the level of FBW7. PLK1 inhibition increased the protein level of endogenous FBW7. Thus, the combinatorial treatment with BI6727 and paclitaxel had a strong impact to increase the endogenous level of the tumor suppressor FBW7.

Although point mutations are rare in ovarian tumors, a hallmark of this cancer type is the mutation of the tumor suppressor P53 with over 94% in HGSOC [53], which could correlate with a loss of the tumor suppression function of P53, including cell cycle inhibition, apoptosis, senescence, DNA repair and autophagy, as well as processes that oppose oncogenic metabolic reprogramming [54]. The mutation of P53 which results in loss of these tumor suppressive functions, increases transcript and protein levels of Aurora A and PLK1 [55] making PLK1 an attractive target for cancers with *TP53* mutation like HGSOC. By analyses of ‘The Cancer Genome Atlas’ datasets, PLK1 expression levels were found to be significantly higher in P53-mutated than in P53–wild-type cancers [56], which also correlates to the observation that the FoxM1 transcription factor network that regulates PLK1 expression was found to be upregulated in 87% of patients with HGSOC [57]. FBW7 expression was found to be negatively correlated with the malignant potential of ovarian tumors [58]. That is to say, FBW7 expression levels in ovarian cancer samples were significantly lower than those in borderline and benign tumors (*P* < 0.01). This study suggests that *TP53* mutations might suppress FBW7 expression through DNA hypermethylation of FBW7 5′-upstream regions. Thus, FBW7 expression was found to be downregulated in ovarian cancers and was associated with P53 mutations and the DNA methylation status of the 5′-upstream regions of FBW7. By means of inhibiting the catalytic activity of PLK1, we were able to stabilize the endogenous level of FBW7, which was associated in combinatorial experiments with a strong downregulation of the critical mitotic timer MCL-1 and the oncogenic driver Cyclin E (Supplementary Figure 9). Consequently, we could enhance the sensitivity of HGSOC to paclitaxel by inhibiting PLK1 activity. In an ovarian cancer cell line without *CCNE1*-amplification, Ovsaho cells, a synergy of BI6727- and paclitaxel-treatment was not observed which might suggest that the preservation of endogenous FBW7 levels leading to downregulation of endogenous Cyclin E is an attractive strategy to sensitize ovarian cancer cells with *CCNE1*-amplification. To date, there have been no clinical trials in which patients were selected based on *CCNE1*-amplification status, and unfortunately, there are no currently available drugs which specifically target Cyclin E1. Hence, several alternative approaches have been proposed to decrease the viability of the *CCNE1*-amplified subset of cancer cells, including the use of several CDK inhibitors [59] and the proteasome inhibitor Bortezomib [27]. Thus, targeting Cyclin E1 by a PLK1 inhibition-based stabilization of FBW7 might be a novel approach to increase apoptosis in *CCNE1*-amplified ovarian cancer cells.

PLK1 holds great promise as therapeutic target. Promising results for the treatment of cultured cancer cells and preclinical trials were reported for Volasertib monotherapy [60]. Volasertib (BI6727) is under current investigation in multiple ongoing clinical trials [61, 62]. However, various lines of evidence from clinical trials revealed that Volasertib alone shows different degrees of efficacy, and only a fraction of patients responds well to single-agent Volasertib suggesting that better patient stratifications and optimized treatment regimens are urgently needed. Here, we have identified a pharmacological interaction between Volasertib and paclitaxel, arguing that prolonging mitotic arrest associated with a stimulation of pro-apoptotic pathways and a downregulation of the oncogenic driver Cyclin E may serve as a useful strategy in ovarian cancer cells with *CCNE1*-amplification.

## MATERIALS AND METHODS

Experimental details on patient samples, genomic profiling, high throughput siRNA transfection, antibodies and chemicals, western blot analysis, colony formation assays and neurite outgrowth assays are given in Supplementary Information.

### Cell culture

The ovarian carcinoma cell lines OVCAR-3 Ovsaho were cultured in RPMI 1640 (Gibco) and COV318 in DMEM (Gibco), respectively, both containing 10% FCS (Gibco) and 1% Penicillin/Streptomycin (Sigma-Aldrich). COV318 cells needed additional 2 mM glutamate (Sigma-Aldrich). PC-12 cells were cultured in DMEM GlutaMAX (Gibco) supplied with 10% FCS, 5% horse serum (Gibco) and 1% Penicillin/Streptomycin.

Primary cells were isolated from HGSOC using the tumor dissociation kit (Max Miltenyi 130-095-929) together with the tumor cell isolation kit (Max Miltenyi 130-108-339) following the manufacturer's instructions. Isolated cells were cultured as described [63] and subsequently adapted to normal cell culture medium containing 10% FCS without growth supplements.

### 3-D cultures

A cell suspension of 3000 cells/ 50 ul was prepared and pipetted from the topside into a 96 well Perfect 3D Hanging Drop plate (BioTrend). Plates were incubated at 37^°^C for several days until hanging drops have developed. The 3-D culture was harvested on a 96 well plate covered with 1% agarose by low spin centrifugation. Treatment of cells with BI6727 and paclitaxel was performed as indicated for 7 to 14 days. Cells were stained with the LIVE/DEAD viability/ cytotoxicity kit (Molecular Probes/Thermofisher) for 30 min and inspected using a fluorescence microscope. The polyanionic dye calcein is retained in live cells, producing an intense uniform green fluorescence, while EthD-1 enters cells with damaged membranes thereby producing a bright red fluorescence upon binding to nucleic acids in dead cells. The ratio of viable/dead cells were calculated with the software ImageJ Fiji.

### Cell proliferation and Caspase-3/7-activity (multiplexed protocol)

48 h following transfection 7 μl substrate of the Cell Titer-Blue^®^ Cell Viability Assay (Promega) were added to each well. After a short centrifugation step (1,000 rpm for 10 sec) cells were incubated for further 3 h at 37°C / 5% CO_2_ before fluorescence reading (Victor X4, PerkinElmer). The activity of Caspase-3/7 was determined using the Caspase-Glo^®^ 3/7 Assay (Promega). 20 μl substrate per well were applied and after 30 minutes shaking at room temperature in the dark, luminescence was detected (Victor X4, Perkin Elmer). The analysis of data was partially done online by Networkanalyst and GraphPad Prism.

### Cell cycle and apoptosis assays

Paclitaxel and/or BI6727 treatment was conducted at least overnight followed by cell cycle and apoptosis measurements after predefined time intervals. For cell cycle analysis, cells were harvested, washed, fixed with 70% EtOH and stained as described [10]. Cell cycle quantification was performed using a FACS Calibur and Cellquest Pro software (both BD Biosciences). The activity of Caspase-3/7 was determined in cell lysates using the Caspase-Glo^®^3/7 Assay according to the manufacturer's instructions. Apoptotic loss of membrane asymmetry was analyzed by staining for OVCAR-3 cells (BD Biosciences) on a FACS Calibur.

### Proliferation assays

To measure cell proliferation using the Cell Titer-Blue^®^ Cell Viability Assay, 2500 cells per well were seeded in 96-well plates and treated with paclitaxel, BI6727 or both. Cells were incubated with 20 μl substrate of the Cell Titer-Blue^®^ Cell Viability Assay for 4 h and the light absorbance was measured at 540 nm (Victor X4, Perkin Elmer). The 50% growth inhibitory concentration (GI 50) was estimated using the following formula: 100 x (T – T0)/(C – T0) = 50, where T is the optical density (OD) value after drug treatment, T0 is the OD value at time 0, and C is the OD value for the diluent treatment [64]. Time 0 was defined as the day the drug was administered.

### Statistical analysis

All experiments were performed at least three times and displayed as mean and standard error of the mean. The statistical significance was assessed by Student's *t*-test (two-tailed and paired) using Excel 2010 (Microsoft) as well as GraphPad Prism 7 (GraphPad, La Jolla, CA, USA). Significant differences (^*^P≤0.05; ^**^P≤0.01; ^***^P≤0.001) are indicated in the figures with asterisks.

### Image work

Images were opened in Adobe Photoshop CS6, sized and placed in figures using Adobe Illustrator CS6 (Adobe Systems, Mountain View, CA).

## SUPPLEMENTARY MATERIALS FIGURES AND TABLE





## Abbreviations

CI: combination index
FACS: flow cytometry
FCS: fetal bovine serum
GI: growth inhibition
HGSOC: high grade serous ovarian carcinoma
IC: inhibitory concentration
Pac: paclitaxel
pH3: phospho-histone H3
PLK1: Polo-like kinase
siRNA: small interfering RNA
SCF: complex of SKP1, CUL1 and F-box protein
