# Supplementary material for: Synthetic lethality in CCNE1-amplified high grade serous ovarian cancer through combined inhibition of Polo-like kinase 1 and microtubule dynamics
Source: Oncotarget. 2018 May 25;9(40):25842–59. doi: 10.18632/oncotarget.25386 (PMC5995225; doi:10.18632/oncotarget.25386)
Supplement: Supplementary file 1 [file oncotarget-09-25842-s001.pdf]

Synthetic lethality in *CCNE1*-amplified high grade serous ovarian cancer through combined inhibition of Polo-like kinase 1 and microtubule dynamics

SUPPLEMENTARY MATERIALS

MATERIALS AND METHODS

Patients and samples

This study was conducted according to the 'REporting recommendations for tumor MARKer prognostic studies' (REMARK) [1]. To establish primary, patient derived ovarian cancer cell cultures, we analyzed samples derived from patients undergoing surgical resection between January 2015 and September 2017 at the Department of Gynecology of the Goethe University Hospital in Frankfurt am Main, Germany. For the samples with validated diagnosis sufficient archival material for immunohistochemical analysis was available. The Local Research Ethics Committees approved studies of human tissue and samples were processed anonymously.

Genomic profiling using the illumina human HT-12 expression BeadChip V4

Total RNA was isolated using the RNeasy Mini kit (Qiagen) following the manufacturer's instructions. 1 µg RNA was amplified and labeled with an Illumina TotalPrep RNA amplification kit (Life Technologies, Ambion), and hybridized according to the manufacturer's protocol (Illumina GeneExpression Direct Hyb; illumina, San Diego, USA). Arrays were read by an Illumina Bead Chip Reader, and data were normalized by quantile normalization in GenePattern (http:// genepattern.broadinstitute.org/gp/pages/index.jsf). The ratios of gene expression in OVCAR-3 cells treated for 24 h with paclitaxel versus untreated cells were determined, and the Top 30 up- and downregulated kinases were tabulated.

High-throughput siRNA-transfection

The siRNA library targeting 711 human kinases (4 siRNAs/well/gene) was purchased from Dharmacon. OVCAR-3 cells were transfected by the reverse method (simultaneous seeding and siRNA-transfection). Briefly, Lipofectamine RNAiMAX (Invitrogen) was diluted in serum-reduced medium (Opti-MEM, Invitrogen) and plated in white, clear-bottom half-area 96 well-plates (Greiner Bio-One). siRNAs were pre-diluted in siRNA-Dilution buffer (Dharmacon) and added to a final concentration of 20 nM. Plates containing the siRNA-lipid mix were spun down (1,000 rpm for 10 seconds) and complexes were allowed to form for 20 min at room temperature. Meanwhile OVCAR-3 cells were trypsinized, collected in fresh medium and counted. Cell suspension (2.100 cells / 40 µL) was prepared in RPMI completed with 12.5% FCS and added to a final volume of 50 µL per well. Each plate included the following controls: Medium alone (background), untreated cells (basal level), 20 nM siGlo Red Transfection Indicator (transfection control, Dharmacon), 20 nM siPLK1 (test efficiency and consistency, Sigma) and 1 µM Campthotecin (apoptosis, cell death and consistency, Enzo Life Sciences). Each plate was analyzed in triplicate.

Antibodies and chemicals

Primary antibodies were obtained from the following sources: PLK1 (05-844), phospho-Histone H3 (Ser10) (05806) from Millipore; Cyclin B1 (GNS1) from Santa Cruz; PARP (9542), phospho-Aurora B (2914), BCL-X_L (2762), Caspase-3 (9668) and Cyclin E (4129) from Cell Signaling; BAK (AMO3-100UG), BAX (B8429) and MCL-1 (ADI-AAP-240-D) from Enzo, Securin (ab3305), pBCL-X_L S62 (ab30655) and EndoG (ab9647) from Abcam, FBW7 (40-1500) Invitrogen, β-Actin (A2228-100UL) from Sigma-Aldrich served as loading control.

Secondary antibodies for western blot analysis against Rabbit (NA934V) and Mouse (NXA931) IgG were obtained from GE Healthcare. Secondary antibodies used for immunofluorescence staining were obtained from Dako (F0313). Paclitaxel (T7402) was purchased from Sigma-Aldrich, BI6727 (BYT-ORB181049) Selleckchem, propidium iodide (440300250) Acros Organics, RNase A (1007885) Qiagen, PE Annexin V (556421), 7AAD (21-68981E) BD Biosciences and β-Nerve-growth factor (G514A) Promega.

Phospho-histone H3 (Ser10) staining

Cells were treated with paclitaxel, BI6727 or both, trypsinized, fixed using 4% paraform-aldehyde (PFA, Sigma Aldrich) in PBS containing 0.01% Triton-X-100 and washed with PBS-T before adding a phospho-Histone H3 (Ser10) specific antibody. Staining was performed

for 1 h at 37°C. Secondary staining was done for 30 min using a FITC-coupled antibody. Fractions of mitotic cells were quantified using a FACS Calibur and Cellquest Pro software (both BD Biosciences).

Western blot analysis

Protein extracts of cells were prepared by lysis in RIPA buffer (Sigma) supplemented with protease inhibitors (Complete protease inhibitor cocktail, Roche). Protein extracts (25 µg) were separated by SDS-PAGE and transferred onto PVDF membranes using the TransBlot Turbo Transfer System (BioRad). After blocking with 5% BSA in PBS with 0.1% Tween-20 for 30 min, the membrane was incubated with primary antibodies for 1 h at room temperature. HRP-linked secondary antibodies were incubated 30 min at room temperature followed by ECL detection (ECL Chemiluminescent Western Blot Substrate, Pierce).

Membrane potential

OVCAR-3 cells were trypsinized, washed in PBS and centrifuged at 700g for 5 min. The cell pellets were incubated with 10 nM $DiOC_6(3)$ in fresh medium at 37°C for 30 min in the dark. After incubation, cells were washed twice and resuspended in PBS and assayed using flow cytometry. The fluorescence intensity of $DiOC_6(3)$ was measured and calculated by BD FACS CellQuest Pro software.

Colony formation assay

Cells were treated with paclitaxel, BI6727 or both overnight followed by seeding predefined numbers in 6-well plates. Colonies were fixed using 70% EtOH and stained with Coomassie Brilliant Blue. The numbers of grown colonies were counted and images were taken using AxioObserver Z1 microscope (Zeiss) as well as the ChemiDoc MP system (BioRad).

Nerve growth factor (NGF)-induced neurite outgrowth assay.

Undifferentiated PC-12 cells were pretreated for 5 days by adding β-NGF (NGF from mouse source) (Promega) to a final concentration of 50 ng/ml, trypsinized, washed using phosphate-buffered saline (PBS), centrifuged and plated in 12-well culture chambers (Costar, Nuclepore). Optimal adherence and neurite formation required precoating the cell culture plastic using collagen I (Gibco), followed by washing with PBS. Differentiated PC-12 cells were co-incubated with paclitaxel, BI6727 or both together with 10 ng/ml β-NGF. Subsequently, cells expressing neuronal like structures were examined using an AxioObserver Z1 microscope (Zeiss). Images were taken using an AxioCam MRm camera and analyzed using ImageJ Fiji.

To discriminate specific neurotoxicity from general cytotoxicity, the analysis was performed on the basis of the fraction of neurite-forming cells instead of the absolute number of cells. The results, therefore, are given as percentage of cells expressing neurites.

Combination index

Measured effects were classified by calculating the combination index as follows: c.i.$(Am)_{50}/(As)_{50}+(Bm)_{50}/(Bs)_{50}$. The variables $(Am)_{50}/(Bm)_{50}$ typify the concentrations of drug A/B achieving 50% growth inhibition in combinatorial treatment (IC_{50}). $(As)_{50}/(Bs)_{50}$ symbolize the concentrations of drug A/B necessary to achieve 50% growth inhibition in single administration. A combination index >1 indicates an antagonistic effect, whereas an index =1 displays an additive and an index <1 a synergistic effect [2].

REFERENCES

1. McShane LM, Altman DG, Sauerbrei W, Taube SE, Gion M, Clark GM; Statistics Subcommittee of NCI-EORTC Working Group on Cancer Diagnostics. REporting recommendations for tumor MARKer prognostic studies (REMARK). Nat Clin Pract Urol. 2005; 2:416-22.
2. Dai D, Holmes AM, Nguyen T, Davies S, Theele DP, Verschraegen C, Leslie KK. A potential synergistic anticancer effect of paclitaxel and amifostine on endometrial cancer. Cancer Res. 2005; 65:9517-24.

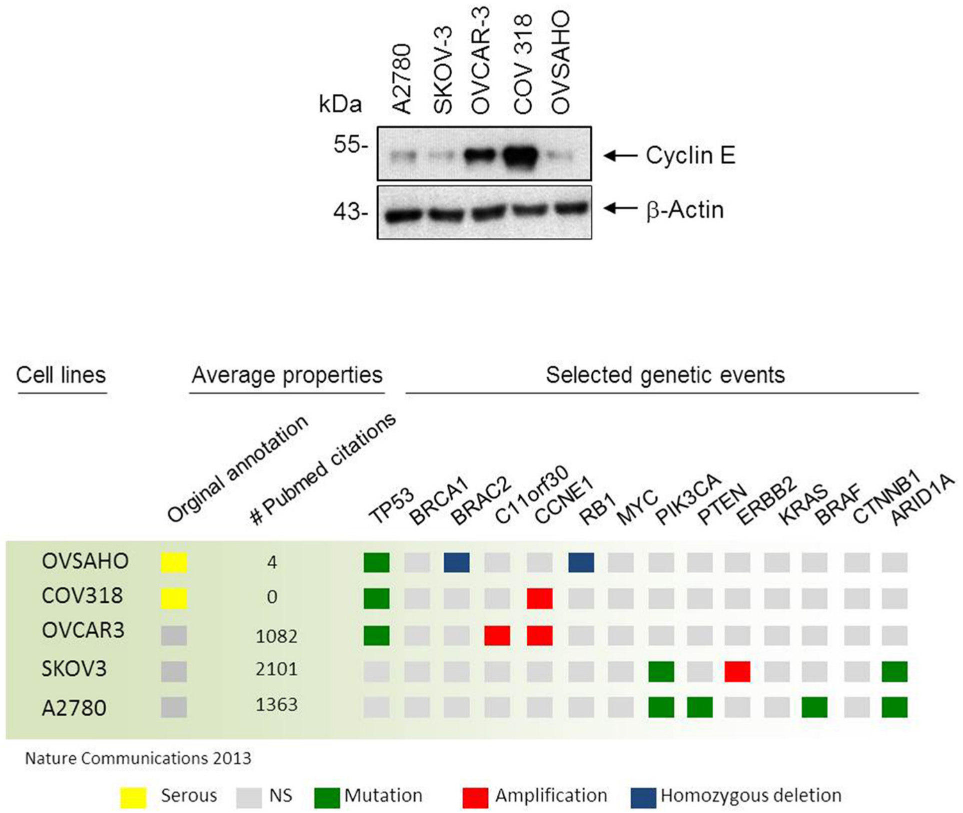

Supplementary Figure 1: Cyclin E expression in ovarian cancer cells. (upper panel) Expression of Cyclin E was analyzed by western blotting. β-Actin served as controls. (lower panel) Genomic profiles of ovarian cancer cells according to Domcke, S. et al.

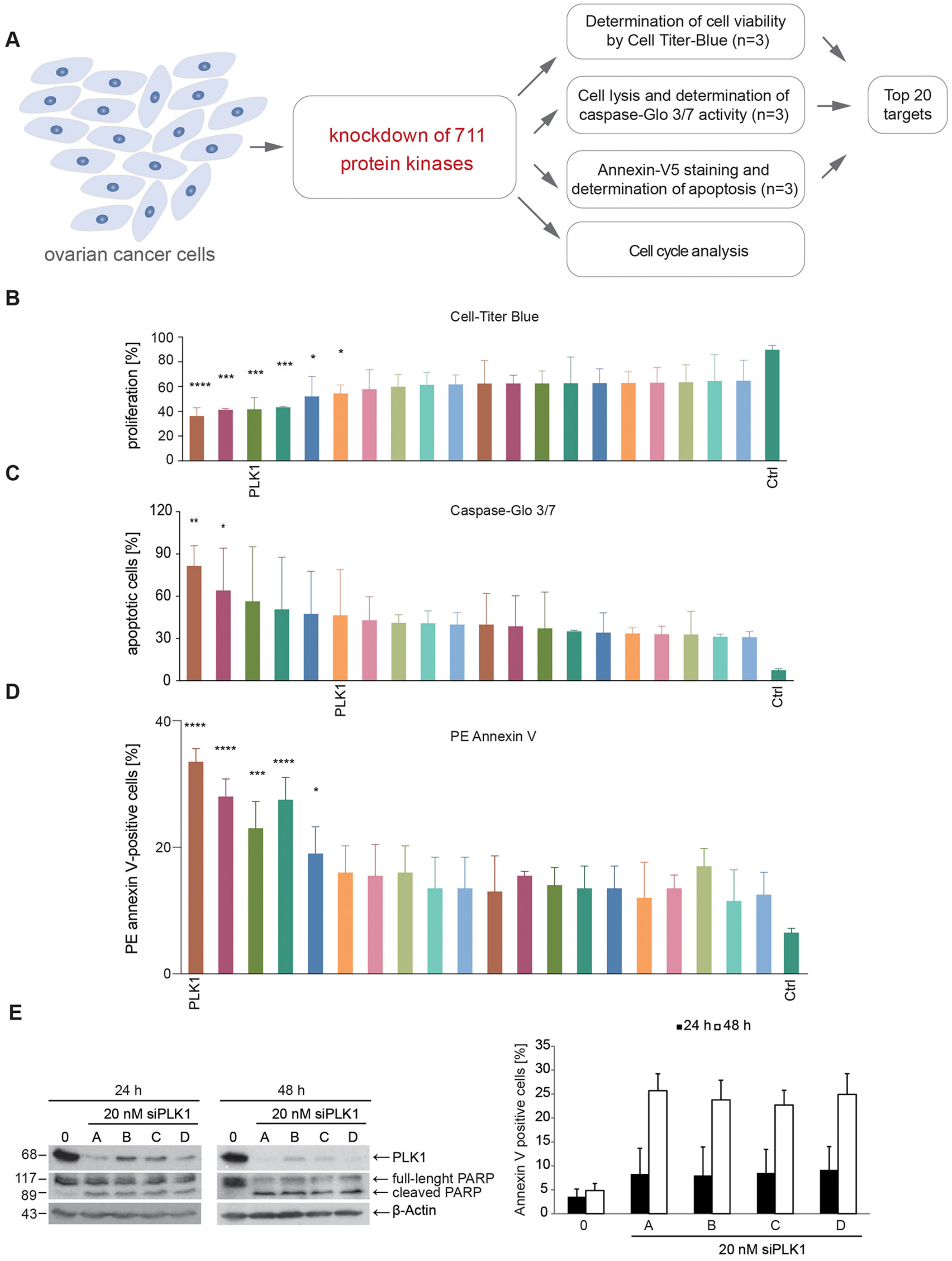

Supplementary Figure 2: Depletion of kinases in HGSOC cells with *CCNE1*-amplification by using the Dharmacon kinome library. **(A)** OVCAR-3 cells were transfected with siRNAs targeting 711 kinases and the effects on **(B)** cell viability using the Cell Titer-Blue® Cell Viability Assay, **(C)** Caspase-3/7 activity using the Caspase-Glo® 3/7 Assay and **(D)** apoptosis induction based on PE Annexin V staining were monitored. **(B-D)** The Top 20-kinase panel was determined. **(E)** PLK1 depletion was validated using single siRNAs (cherry picking) that made up the pool in the primary screening. PE Annexin V staining was monitored.

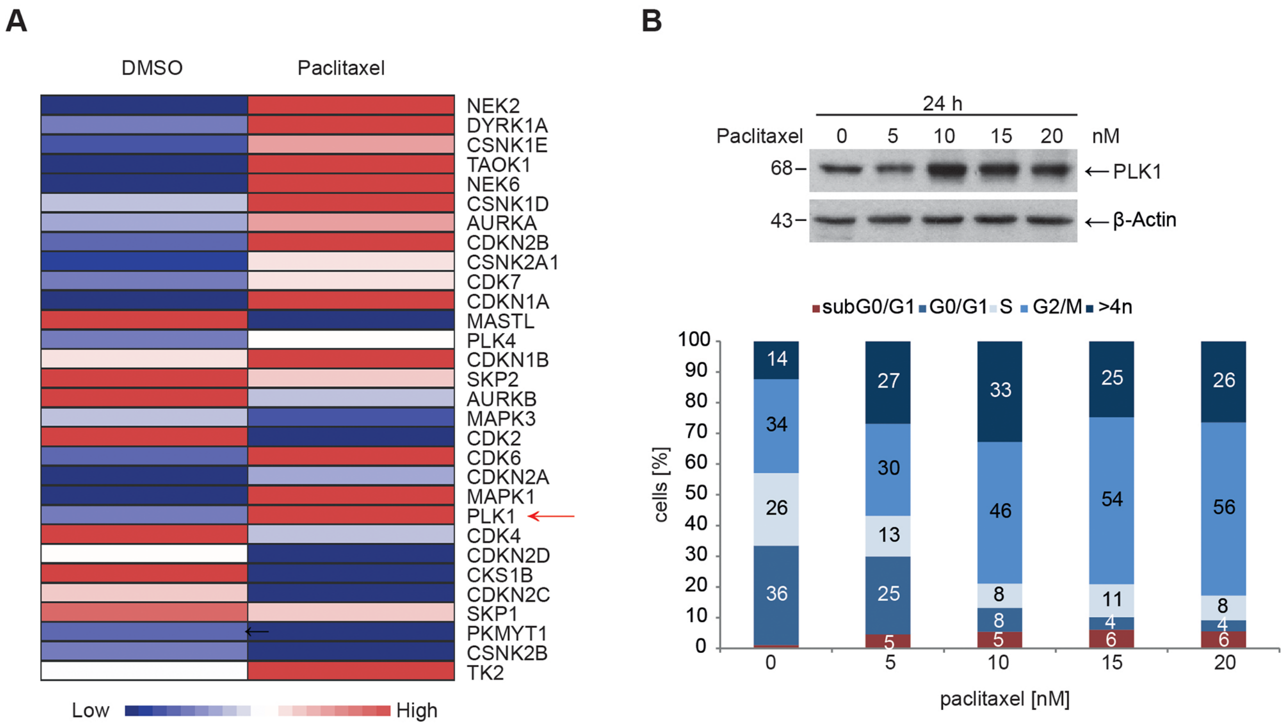

Supplementary Figure 3: Paclitaxel-treatment arrests cells in mitosis associated with elevated levels of PLK1 expression. (A) Whole cell RNA extracts of low dose paclitaxel-treated OVCAR-3 cells were examined by cDNA array analysis, Top 30-kinases giving the highest expression differences compared to control cells are shown. **(B)** OVCAR-3 cells were treated with increasing paclitaxel concentrations for 24 h. PLK1 expression was evaluated by immunoblotting using PLK1 antibodies (upper panel) and cell cycle distribution was analyzed using flow cytometry (lower panel).

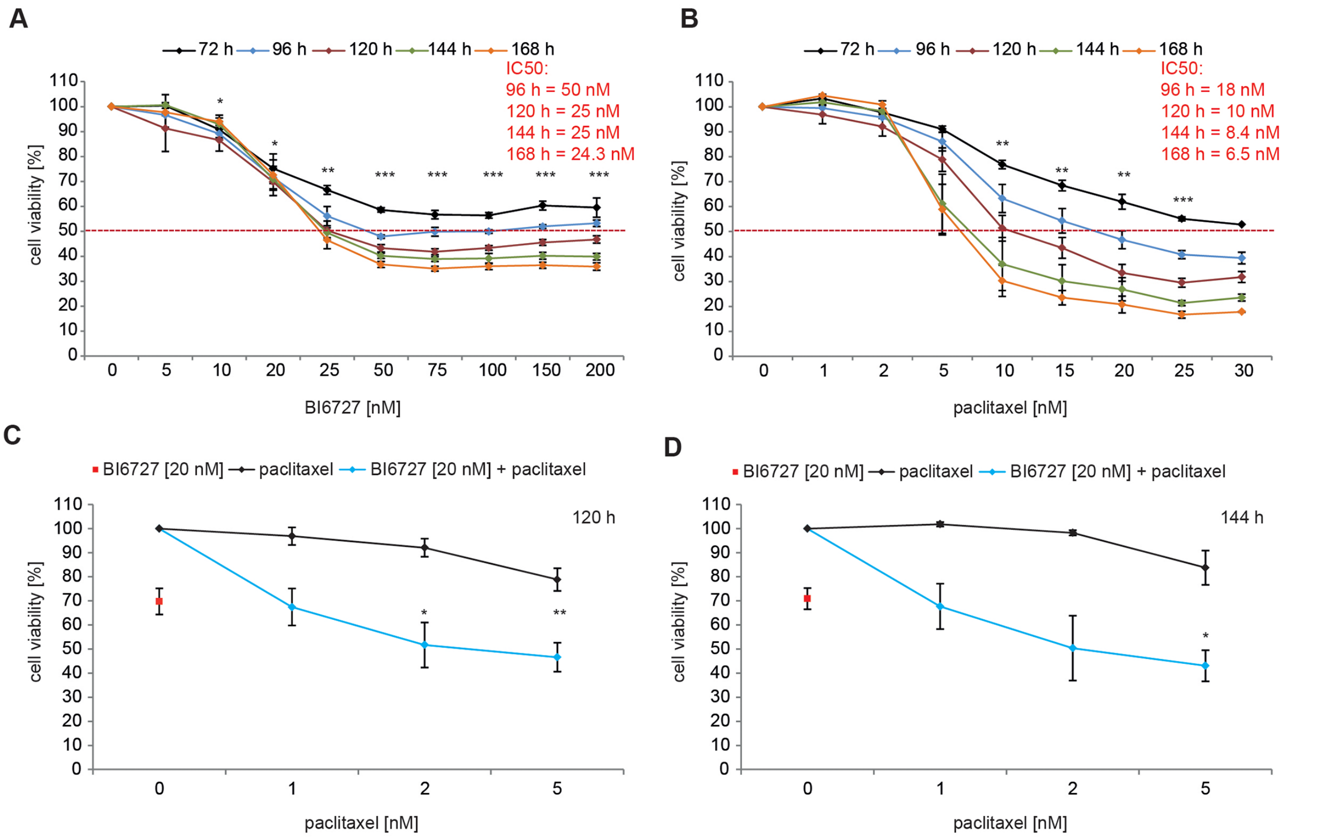

Supplementary Figure 4: BI6727 treatment sensitizes HGSOC cells COV318 to paclitaxel. (A) COV318 cells were treated with increasing concentrations of BI6727 or **(B)** paclitaxel for 7 d. The cell viability was measured using the Cell Titer-Blue® Cell Viability Assay. **(C, D)** COV318 cells were treated with either 20 nM BI6727 or increasing paclitaxel concentrations or both for up to 6 d. The cell viability was measured. Measurements were statistically significant by two-tailed Student's *t*-test ($^{*}P \leq 0.05$; $^{**}P \leq 0.01$). Each measurement represents the mean value ± SEM (n=3).

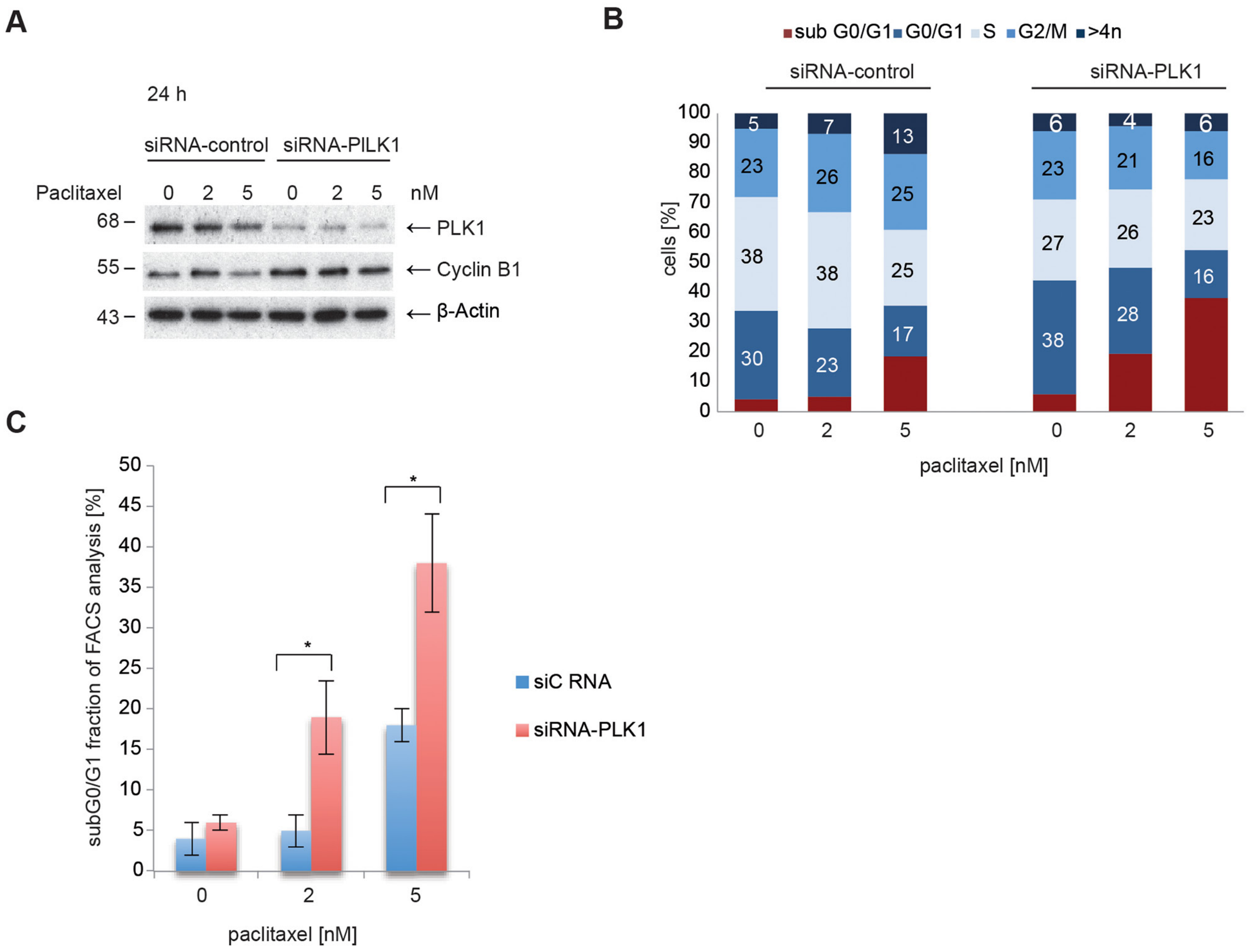

Supplementary Figure 5: The knockdown of PLK1 by RNAi sensitizes ovarian cancer cells to paclitaxel treatment. (A) OVCAR-3 cells were transfected with either siRNA against PLK1 or control siRNA and treated with increasing paclitaxel concentrations. Knockdown of PLK1 was analyzed by western blotting. Cyclin B1 and β-Actin served as controls. **(B)** The cell cycle distribution was determined by flow cytometry and PI staining. **(C)** The sub G_0/G_1-fraction as indicator for apoptosis following siRNA-PLK1 transfection and combinatorial paclitaxel treatment is shown.

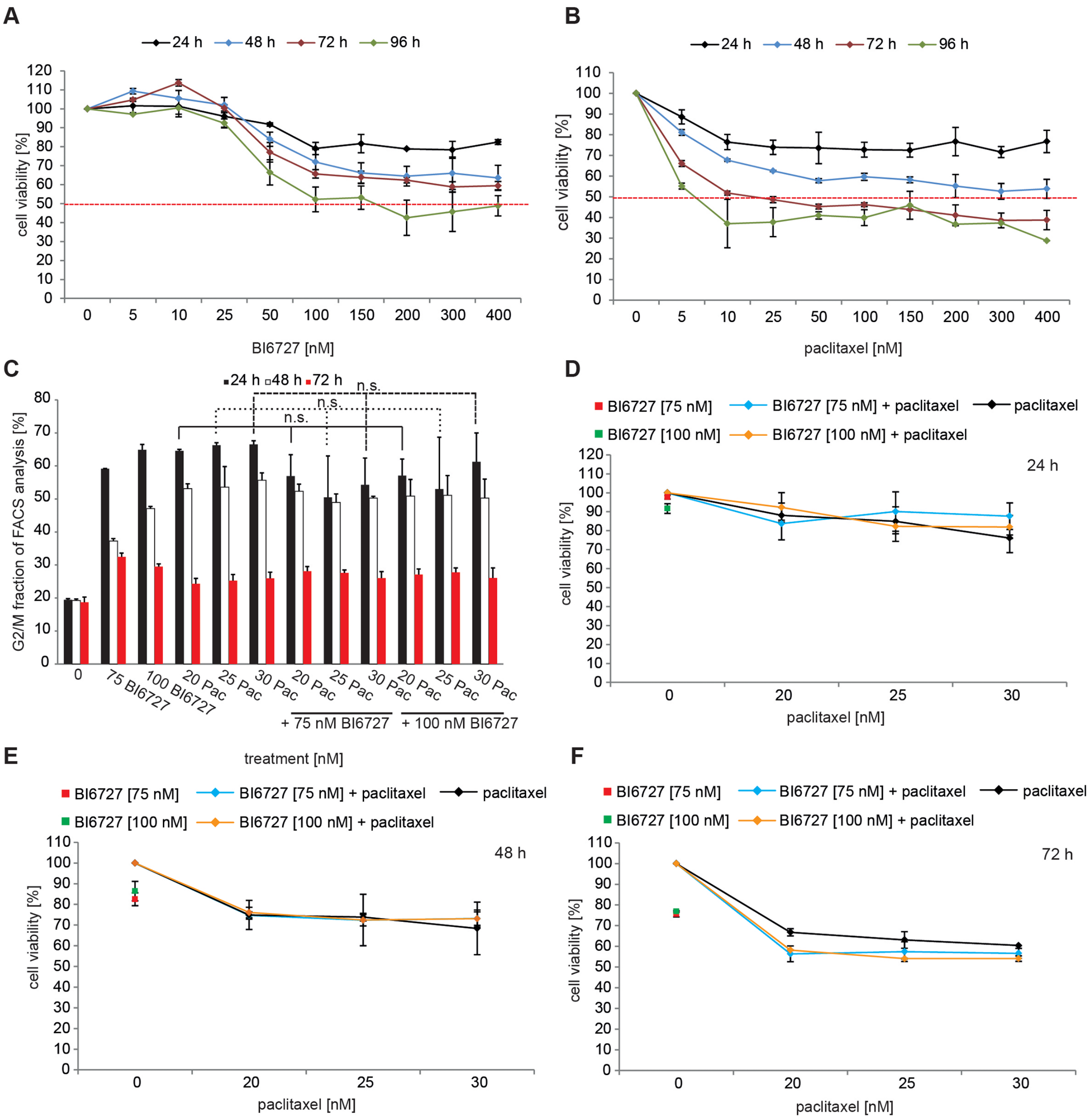

Supplementary Figure 6: Treatment of Ovsaho cells with BI6727 and/or paclitaxel. **(A)** Cells were treated with increasing concentrations of BI6727 or **(B)** paclitaxel for 4 d. The cell viability was measured using the Cell Titer-Blue® Cell Viability Assay. **(C)** The G_2/M fraction was determined over 3 d post-treatment using flow cytometry. Measurements were statistically not significant by two-tailed Student's *t*-test (n.s., not significant). Each bar graph represents the mean value ± SEM (n=3). **(D-F)** Cells were treated with either 75 or 100 nM BI6727, increasing paclitaxel concentrations or both for up to 3 d. The cell viability was measured. Measurements were statistically not significant by two-tailed Student's *t*-test. Each measurement represents the mean value ± SEM (n=3).

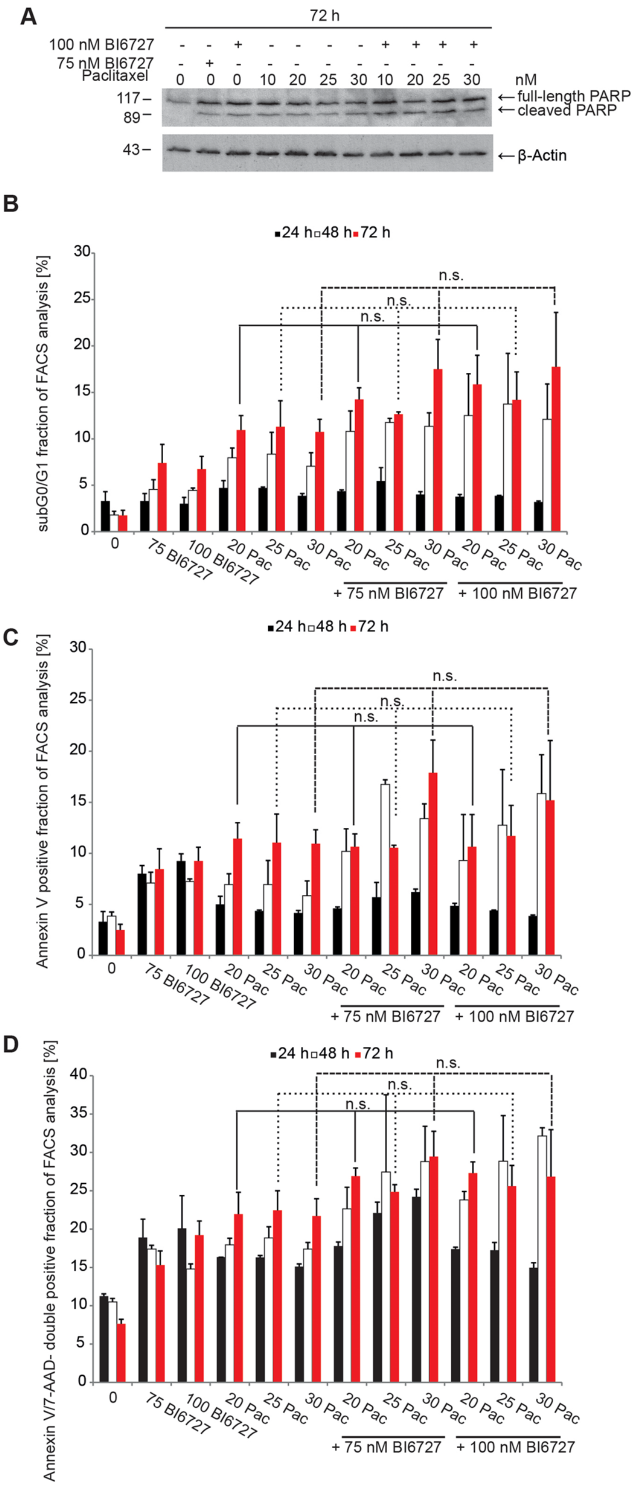

Supplementary Figure 7: Induction of apoptosis in Ovsaho cells upon treatment with BI6727 and/or paclitaxel. (A) Cells were treated with 75 or 100 nM BI6727, increasing paclitaxel concentrations or both. Apoptosis was analyzed 48 h post-treatment by western blotting for full-length and cleaved PARP. β-Actin served as loading control. Apoptosis was validated **(B)** by measuring the sub G_0/G_1 fractions or **(C)** by PE Annexin V staining or **(D)** by PE-Annexin V/7-AAD staining. Measurements were statistically not significant by two-tailed Student's t-test (n.s., not significant). Each bar graph represents the mean value ± SEM (n=3).

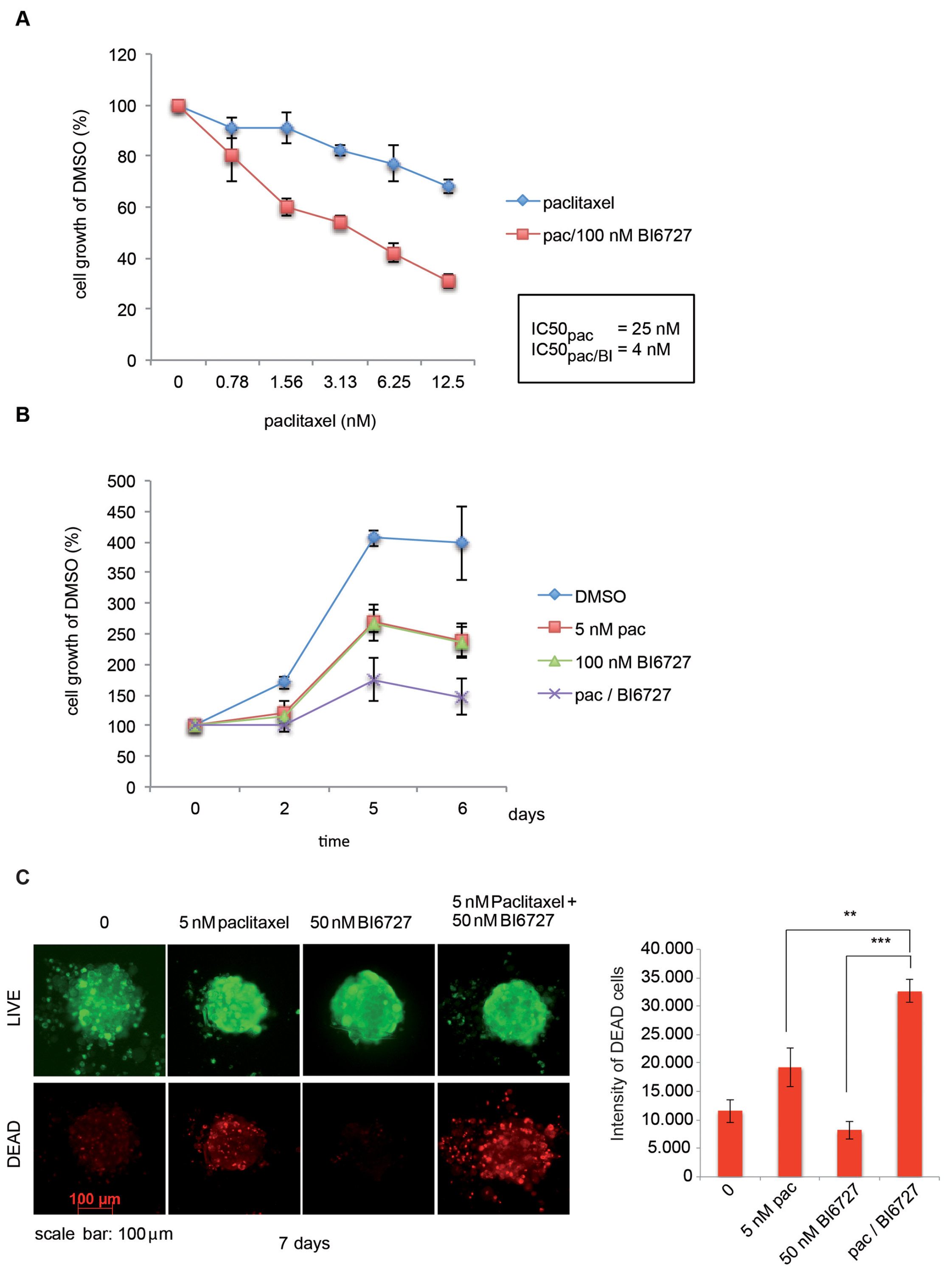

Supplementary Figure 8: BI6727 treatment sensitizes patient-derived HGSOC cells to paclitaxel. **(A)** Primary tumor cells were either treated with increasing paclitaxel concentrations or paclitaxel/100 nM BI6727. The cell viability was determined for 72 h or **(B)** over a period of 6 d using the Cell Titer-Blue Cell® Viability Assay. **(C)** 3-D cultures grown out of primary tumor cells were treated with either 5 nM paclitaxel, 50 nM BI6727 or both for 7 d. Cells were stained and fluorescence intensities of dead cells were determined. Measurements were statistically significant by two-tailed Student's *t*-test ($^{*}P \leq 0.05$; $^{**}P \leq 0.01$). Each bar graph represents the mean value ± SEM (n=3).

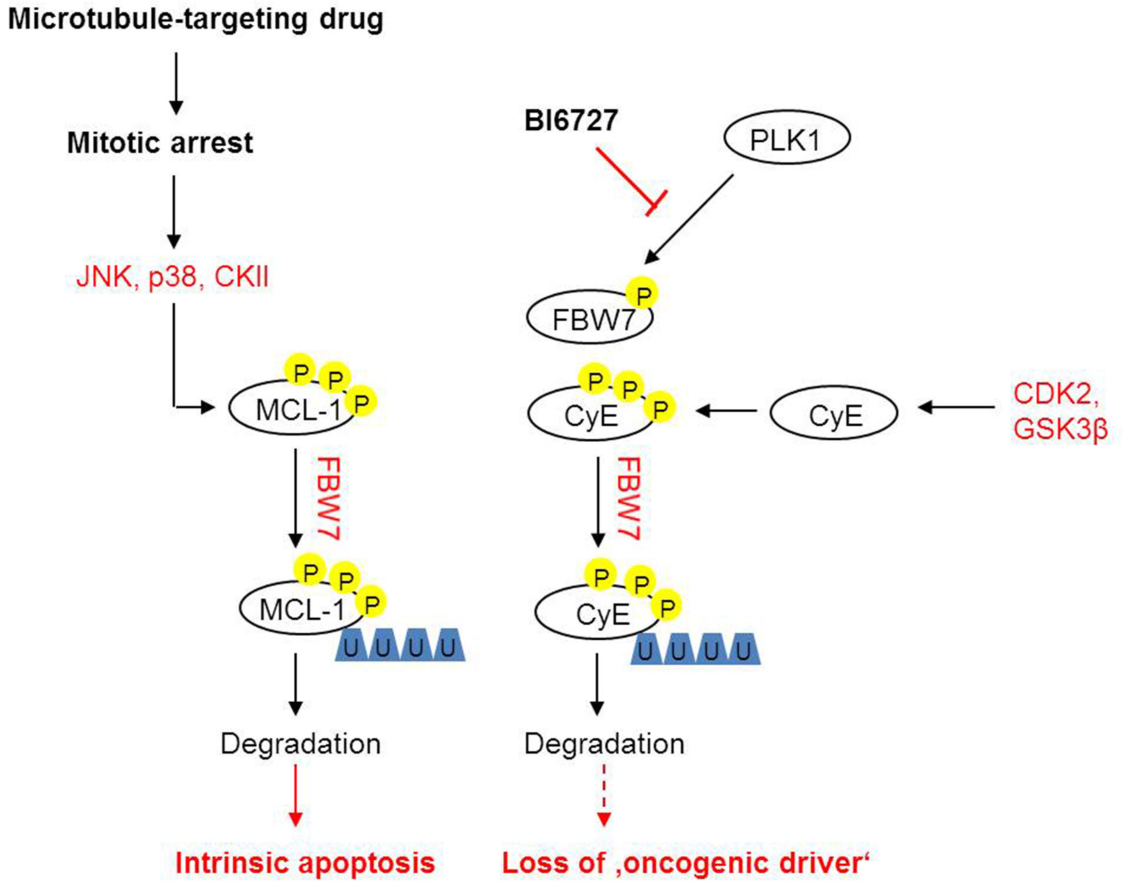

Supplementary Figure 9: Treatment with microtubule-targeting agents like paclitaxel leads to mitotic arrest and triggers the onset of MCL-1 degradation via the SCF^{Fbw7} pathway. MCL-1 is acted on by a set of pro-apoptotic proteins that drive its degradation and a set of pro-survival proteins that enhance its stability. Activated PLK1 phosphorylates FBW7 inducing its degradation. By inhibiting PLK1 the levels of FBW7 increases leading to the downregulation of MCL-1 and Cyclin E, which favors the pro-apoptotic pathway in *CCNE1*-amplified cells during prolonged mitotic arrest induced by paclitaxel and PLK1 inhibition.

Supplementary Table 1: Kinases exhibiting differential expression in paclitaxel-treated OVCAR-3 cells.

See Supplementary File 1
