# Supplementary material for: Synthetic lethality in CCNE1-amplified high grade serous ovarian cancer through combined inhibition of Polo-like kinase 1 and microtubule dynamics
Source: Oncotarget. 2018 May 25;9(40):25842–59. doi: 10.18632/oncotarget.25386 (PMC5995225; doi:10.18632/oncotarget.25386)
Supplement: Supplementary file 2 [file oncotarget-09-25842-s002.docx]

**Supplementary Table 1:** Kinases exhibiting differential expression in paclitaxel-treated cells.

| **Symbols** | **EntrezID** | **logFC** | **AveExpr** | **t** | **P.Value** | **adj.P.Val** | **B** | **Name** |
| --- | --- | --- | --- | --- | --- | --- | --- | --- |
| PTK2 | 5747 | -150,89 | 1063.4 | -14,62 | 5,69E-04 | 0,26 | -4,35 | PTK2 protein tyrosine kinase 2 |
| DYRK2 | 8445 | -94,31 | 390.89 | -10,93 | 1,39E-03 | 0,26 | -4,35 | dual-specificity tyrosine-(Y)-phosphorylation regulated kinase 2 |
| NEK2 | 4751 | -111,47 | 809.84 | -8,80 | 2,68E-03 | 0,29 | -4,36 | NIMA (never in mitosis gene a)-related kinase 2 |
| DYRK1A | 1859 | -80,20 | 361.3 | -8,41 | 3,07E-03 | 0,29 | -4,36 | dual-specificity tyrosine-(Y)-phosphorylation regulated kinase 1A |
| CLK1 | 1195 | -59,99 | 223.25 | -8,38 | 3,10E-03 | 0,29 | -4,36 | CDC-like kinase 1 |
| MAPKAP1 | 79109 | -44,67 | 442.4 | -7,52 | 4,29E-03 | 0,34 | -4,36 | mitogen-activated protein kinase associated protein 1 |
| STK36 | 27148 | -166,13 | 656.26 | -6,88 | 5,60E-03 | 0,35 | -4,36 | serine/threonine kinase 36 |
| CSNK1G1 | 53944 | -40,92 | 296.5 | -6,85 | 5,66E-03 | 0,35 | -4,36 | casein kinase 1 gamma 1 |
| CSNK1E | 1454 | -143,53 | 700.1 | -6,61 | 6,29E-03 | 0,35 | -4,37 | casein kinase 1 epsilon |
| PRKAG2 | 51422 | -135,02 | 478.95 | -6,19 | 7,64E-03 | 0,36 | -4,37 | protein kinase AMP-activated gamma 2 non-catalytic subunit |
| PRKAR1A | 5573 | -172,35 | 961.06 | -6,01 | 8,30E-03 | 0,36 | -4,37 | protein kinase cAMP-dependent regulatory type I alpha (tissue specific extinguisher 1) |
| GSK3B | 2932 | -134,57 | 758.56 | -5,88 | 8,85E-03 | 0,36 | -4,37 | glycogen synthase kinase 3 beta |
| SNRK | 54861 | -37,72 | 330.85 | -5,84 | 9,02E-03 | 0,36 | -4,37 | SNF related kinase |
| ROR1 | 4919 | -32,91 | 197.67 | -5,29 | 1,20E-02 | 0,39 | -4,38 | receptor tyrosine kinase-like orphan receptor 1 |
| RPS6KC1 | 26750 | -31,59 | 212.92 | -5,25 | 1,23E-02 | 0,39 | -4,38 | ribosomal protein S6 kinase 52kDa polypeptide 1 |
| RIPK4 | 54101 | -90,51 | 568.8 | -5,22 | 1,25E-02 | 0,39 | -4,38 | receptor-interacting serine-threonine kinase 4 |
| SGK1 | 6446 | -90,78 | 231.65 | -5,10 | 1,33E-02 | 0,39 | -4,38 | serum/glucocorticoid regulated kinase 1 |
| TAOK1 | 57551 | -66,20 | 235.16 | -5,09 | 1,34E-02 | 0,39 | -4,38 | TAO kinase 1 |
| NEK6 | 10783 | -48,73 | 229.19 | -4,85 | 1,53E-02 | 0,43 | -4,38 | NIMA (never in mitosis gene a)-related kinase 6 |
| SMG1 | 23049 | -113,27 | 344.2 | -4,77 | 1,62E-02 | 0,43 | -4,39 | smg-1 homolog phosphatidylinositol 3-kinase-related kinase (C. elegans) |
| CSNK1D | 1453 | -92,40 | 741.99 | -4,69 | 1,69E-02 | 0,43 | -4,39 | casein kinase 1 delta |
| CKS2 | 1164 | -714,98 | 3297.7 | -4,55 | 1,84E-02 | 0,44 | -4,39 | CDC28 protein kinase regulatory subunit 2 |
| PMVK | 10654 | -68,75 | 336.78 | -4,43 | 1,98E-02 | 0,44 | -4,39 | phosphomevalonate kinase |
| ULK1 | 8408 | -84,74 | 599.95 | -4,42 | 1,99E-02 | 0,44 | -4,39 | unc-51-like kinase 1 (C. elegans) |
| UHMK1 | 127933 | -26,42 | 165.4 | -4,23 | 2,24E-02 | 0,44 | -4,40 | U2AF homology motif (UHM) kinase 1 |
| PIP5K1A | 8394 | -25,24 | 140.28 | -4,23 | 2,25E-02 | 0,44 | -4,40 | phosphatidylinositol-4-phosphate 5-kinase type I alpha |
| AURKA | 6790 | -643,43 | 2759.7 | -4,18 | 2,32E-02 | 0,44 | -4,40 | aurora kinase A |
| MAP2K1 | 5604 | 47,72 | 815.99 | 4,17 | 2,33E-02 | 0,44 | -4,40 | mitogen-activated protein kinase kinase 1 |
| CDKN3 | 1033 | -456,72 | 1806.8 | -4,07 | 2,50E-02 | 0,44 | -4,40 | cyclin-dependent kinase inhibitor 3 |
| SPHK2 | 56848 | -86,68 | 805.57 | -3,91 | 2,77E-02 | 0,44 | -4,40 | sphingosine kinase 2 |
| CASK | 8573 | -24,08 | 187.61 | -3,86 | 2,88E-02 | 0,44 | -4,40 | calcium/calmodulin-dependent serine protein kinase (MAGUK family) |
| EEF2K | 29904 | -37,71 | 487.68 | -3,84 | 2,92E-02 | 0,44 | -4,40 | eukaryotic elongation factor-2 kinase |
| DGUOK | 1716 | -109,34 | 1520.9 | -3,82 | 2,96E-02 | 0,44 | -4,41 | deoxyguanosine kinase |
| CDKN2B | 1030 | -85,88 | 253.65 | -3,79 | 3,03E-02 | 0,44 | -4,41 | cyclin-dependent kinase inhibitor 2B (p15 inhibits CDK4) |
| PANK4 | 55229 | -26,75 | 163.15 | -3,77 | 3,06E-02 | 0,44 | -4,41 | pantothenate kinase 4 |
| DSTYK | 25778 | -22,50 | 153.59 | -3,72 | 3,19E-02 | 0,44 | -4,41 | dual serine/threonine and tyrosine protein kinase |
| CSNK2A1 | 1457 | -47,75 | 249.95 | -3,69 | 3,25E-02 | 0,44 | -4,41 | casein kinase 2 alpha 1 polypeptide |
| ILK | 3611 | -156,12 | 791.61 | -3,60 | 3,47E-02 | 0,44 | -4,41 | integrin-linked kinase |
| CDK7 | 1022 | -323,31 | 1078 | -3,59 | 3,50E-02 | 0,44 | -4,41 | cyclin-dependent kinase 7 |
| TESK1 | 7016 | 34,79 | 273.81 | 3,58 | 3,51E-02 | 0,44 | -4,41 | testis-specific kinase 1 |
| ROCK2 | 9475 | -985,36 | 4671.4 | -3,58 | 3,51E-02 | 0,44 | -4,41 | Rho-associated coiled-coil containing protein kinase 2 |
| RIOK1 | 83732 | -32,40 | 213.87 | -3,56 | 3,57E-02 | 0,44 | -4,41 | RIO kinase 1 (yeast) |
| CDK10 | 8558 | -31,15 | 277.67 | -3,55 | 3,58E-02 | 0,44 | -4,41 | cyclin-dependent kinase 10 |
| RIPK2 | 8767 | -92,88 | 486.62 | -3,55 | 3,59E-02 | 0,44 | -4,41 | receptor-interacting serine-threonine kinase 2 |
| MERTK | 10461 | -21,36 | 146.91 | -3,43 | 3,92E-02 | 0,47 | -4,42 | c-mer proto-oncogene tyrosine kinase |
| CDKN1A | 1026 | -51,92 | 151.96 | -3,42 | 3,95E-02 | 0,47 | -4,42 | cyclin-dependent kinase inhibitor 1A (p21 Cip1) |
| MASTL | 84930 | 19,90 | 203.86 | 3,36 | 4,13E-02 | 0,48 | -4,42 | microtubule associated serine/threonine kinase-like |
| CDKL3 | 51265 | -19,38 | 146.48 | -3,29 | 4,38E-02 | 0,49 | -4,42 | cyclin-dependent kinase-like 3 |
| EIF2AK4 | 440275 | -80,59 | 265.86 | -3,20 | 4,68E-02 | 0,49 | -4,43 | eukaryotic translation initiation factor 2 alpha kinase 4 |
| MAPK8IP1 | 9479 | -21,55 | 123.17 | -3,17 | 4,80E-02 | 0,49 | -4,43 | mitogen-activated protein kinase 8 interacting protein 1 |
| PACSIN3 | 29763 | 31,29 | 283.75 | 3,16 | 4,85E-02 | 0,49 | -4,43 | protein kinase C and casein kinase substrate in neurons 3 |
| SH3KBP1 | 30011 | -37,22 | 510.38 | -3,13 | 4,94E-02 | 0,49 | -4,43 | SH3-domain kinase binding protein 1 |
| IRAK3 | 11213 | -21,21 | 119.71 | -3,11 | 5,03E-02 | 0,49 | -4,43 | interleukin-1 receptor-associated kinase 3 |
| CSNK1G3 | 1456 | -34,56 | 217.47 | -3,10 | 5,07E-02 | 0,49 | -4,43 | casein kinase 1 gamma 3 |
| RPS6KB1 | 6198 | -17,50 | 502.27 | -3,07 | 5,21E-02 | 0,49 | -4,43 | ribosomal protein S6 kinase 70kDa polypeptide 1 |
| PIK3R3 | 8503 | -22,30 | 133.17 | -3,06 | 5,23E-02 | 0,49 | -4,43 | phosphoinositide-3-kinase regulatory subunit 3 (gamma) |
| CARKD | 55739 | -34,54 | 408.35 | -3,06 | 5,26E-02 | 0,49 | -4,43 | carbohydrate kinase domain containing |
| PANK3 | 79646 | -22,50 | 159.81 | -3,04 | 5,34E-02 | 0,49 | -4,43 | pantothenate kinase 3 |
| PRKAB1 | 5564 | -34,43 | 332.99 | -2,95 | 5,72E-02 | 0,49 | -4,44 | protein kinase AMP-activated beta 1 non-catalytic subunit |
| HIPK2 | 28996 | -89,32 | 288.05 | -2,95 | 5,75E-02 | 0,49 | -4,44 | homeodomain interacting protein kinase 2 |
| PLK4 | 10733 | -120,87 | 694.67 | -2,95 | 5,76E-02 | 0,49 | -4,44 | polo-like kinase 4 |
| STK33 | 65975 | -20,14 | 156.96 | -2,94 | 5,81E-02 | 0,49 | -4,44 | serine/threonine kinase 33 |
| STK10 | 6793 | -24,30 | 111.36 | -2,93 | 5,83E-02 | 0,49 | -4,44 | serine/threonine kinase 10 |
| RPS6KA5 | 9252 | 30,39 | 150.78 | 2,93 | 5,83E-02 | 0,49 | -4,44 | ribosomal protein S6 kinase 90kDa polypeptide 5 |
| CKB | 1152 | -83,66 | 860.04 | -2,92 | 5,88E-02 | 0,49 | -4,44 | creatine kinase brain |
| AGK | 55750 | -71,37 | 586.59 | -2,92 | 5,90E-02 | 0,49 | -4,44 | acylglycerol kinase |
| PIK3C2A | 5286 | -49,22 | 381.63 | -2,88 | 6,06E-02 | 0,49 | -4,44 | phosphoinositide-3-kinase class 2 alpha polypeptide |
| MAP4K5 | 11183 | -68,33 | 421.33 | -2,84 | 6,28E-02 | 0,50 | -4,44 | mitogen-activated protein kinase kinase kinase kinase 5 |
| MAP4K3 | 8491 | -45,15 | 247.97 | -2,84 | 6,31E-02 | 0,50 | -4,44 | mitogen-activated protein kinase kinase kinase kinase 3 |
| NUAK1 | 9891 | -77,94 | 342.59 | -2,82 | 6,41E-02 | 0,50 | -4,44 | NUAK family SNF1-like kinase 1 |
| PIK3CD | 5293 | 20,82 | 180.11 | 2,77 | 6,66E-02 | 0,50 | -4,44 | phosphoinositide-3-kinase catalytic delta polypeptide |
| AXL | 558 | -30,74 | 204.89 | -2,76 | 6,72E-02 | 0,50 | -4,44 | AXL receptor tyrosine kinase |
| CINP | 51550 | -29,74 | 224.16 | -2,75 | 6,79E-02 | 0,50 | -4,45 | cyclin-dependent kinase 2 interacting protein |
| HK2 | 3099 | -50,16 | 387.79 | -2,72 | 6,98E-02 | 0,50 | -4,45 | hexokinase 2 |
| CALM1 | 801 | -127,91 | 1034.8 | -2,70 | 7,06E-02 | 0,50 | -4,45 | calmodulin 1 (phosphorylase kinase delta) |
| CSNK2A1P | 283106 | -26,73 | 201.8 | -2,67 | 7,30E-02 | 0,50 | -4,45 | casein kinase 2 alpha 1 polypeptide pseudogene |
| FASTK | 10922 | -52,21 | 503.18 | -2,66 | 7,33E-02 | 0,50 | -4,45 | Fas-activated serine/threonine kinase |
| PIK3R1 | 5295 | -16,06 | 150.54 | -2,66 | 7,38E-02 | 0,50 | -4,45 | phosphoinositide-3-kinase regulatory subunit 1 (alpha) |
| MAP2K4 | 6416 | -45,27 | 334.97 | -2,65 | 7,38E-02 | 0,50 | -4,45 | mitogen-activated protein kinase kinase 4 |
| CDKN1B | 1027 | -61,21 | 598.03 | -2,65 | 7,39E-02 | 0,50 | -4,45 | cyclin-dependent kinase inhibitor 1B (p27 Kip1) |
| SKP2 | 6502 | 81,05 | 735.33 | 2,65 | 7,42E-02 | 0,50 | -4,45 | S-phase kinase-associated protein 2 E3 ubiquitin protein ligase |
| MAP3K8 | 1326 | -16,64 | 112.48 | -2,64 | 7,51E-02 | 0,50 | -4,45 | mitogen-activated protein kinase kinase kinase 8 |
| HIPK4 | 147746 | -16,61 | 87.188 | -2,61 | 7,68E-02 | 0,50 | -4,45 | homeodomain interacting protein kinase 4 |
| STK32C | 282974 | -18,21 | 112.83 | -2,59 | 7,82E-02 | 0,50 | -4,45 | serine/threonine kinase 32C |
| TLK2 | 11011 | -33,65 | 318.49 | -2,59 | 7,83E-02 | 0,50 | -4,45 | tousled-like kinase 2 |
| MAP4K4 | 9448 | -16,08 | 167.15 | -2,58 | 7,86E-02 | 0,50 | -4,45 | mitogen-activated protein kinase kinase kinase kinase 4 |
| SLK | 9748 | -28,31 | 292.97 | -2,55 | 8,12E-02 | 0,50 | -4,46 | STE20-like kinase |
| CSNK1A1 | 1452 | -55,32 | 281.51 | -2,53 | 8,21E-02 | 0,50 | -4,46 | casein kinase 1 alpha 1 |
| HGS | 9146 | -226,90 | 2159.4 | -2,53 | 8,22E-02 | 0,50 | -4,46 | hepatocyte growth factor-regulated tyrosine kinase substrate |
| MAPKAPK2 | 9261 | -23,23 | 200.92 | -2,51 | 8,37E-02 | 0,50 | -4,46 | mitogen-activated protein kinase-activated protein kinase 2 |
| PLAU | 5328 | -282,61 | 884.87 | -2,51 | 8,38E-02 | 0,50 | -4,46 | plasminogen activator urokinase |
| AKAP8L | 26993 | -21,86 | 156.36 | -2,49 | 8,54E-02 | 0,50 | -4,46 | A kinase (PRKA) anchor protein 8-like |
| PI4KA | 5297 | -30,29 | 481.78 | -2,49 | 8,56E-02 | 0,50 | -4,46 | phosphatidylinositol 4-kinase catalytic alpha |
| STK19 | 8859 | -22,69 | 399.59 | -2,47 | 8,72E-02 | 0,50 | -4,46 | serine/threonine kinase 19 |
| DOK1 | 1796 | 16,04 | 100.71 | 2,46 | 8,80E-02 | 0,50 | -4,46 | docking protein 1 62kDa (downstream of tyrosine kinase 1) |
| PSTK | 118672 | -23,11 | 147.64 | -2,42 | 9,08E-02 | 0,50 | -4,46 | phosphoseryl-tRNA kinase |
| RIPK1 | 8737 | -27,07 | 193.19 | -2,41 | 9,21E-02 | 0,50 | -4,47 | receptor (TNFRSF)-interacting serine-threonine kinase 1 |
| PI4K2A | 55361 | -14,76 | 132.8 | -2,40 | 9,29E-02 | 0,50 | -4,47 | phosphatidylinositol 4-kinase type 2 alpha |
| MAPKAPK5 | 8550 | -74,81 | 676.56 | -2,39 | 9,37E-02 | 0,50 | -4,47 | mitogen-activated protein kinase-activated protein kinase 5 |
| MAP3K7 | 6885 | -92,44 | 561.86 | -2,38 | 9,45E-02 | 0,50 | -4,47 | mitogen-activated protein kinase kinase kinase 7 |
| AURKB | 9212 | 41,49 | 670.56 | 2,38 | 9,49E-02 | 0,50 | -4,47 | aurora kinase B |
| PRKAG1 | 5571 | -65,41 | 349.42 | -2,36 | 9,63E-02 | 0,50 | -4,47 | protein kinase AMP-activated gamma 1 non-catalytic subunit |
| EIF2AK1 | 27102 | 75,09 | 2473.9 | 2,34 | 9,84E-02 | 0,50 | -4,47 | eukaryotic translation initiation factor 2-alpha kinase 1 |
| CDC42BPA | 8476 | -19,13 | 141.01 | -2,32 | 9,95E-02 | 0,50 | -4,47 | CDC42 binding protein kinase alpha (DMPK-like) |
| NME5 | 8382 | -25,60 | 143.12 | -2,32 | 9,98E-02 | 0,50 | -4,47 | non-metastatic cells 5 protein expressed in (nucleoside-diphosphate kinase) |
| PKN3 | 29941 | 14,37 | 112.46 | 2,32 | 9,98E-02 | 0,50 | -4,47 | protein kinase N3 |
| PKN2 | 5586 | -24,93 | 189.46 | -2,32 | 9,99E-02 | 0,50 | -4,47 | protein kinase N2 |
| MAPK3 | 5595 | 90,40 | 629.58 | 2,32 | 1,00E-01 | 0,50 | -4,47 | mitogen-activated protein kinase 3 |
| PRKACG | 5568 | -17,87 | 94.813 | -2,31 | 1,01E-01 | 0,50 | -4,47 | protein kinase cAMP-dependent catalytic gamma |
| HCK | 3055 | 21,65 | 161.04 | 2,29 | 1,02E-01 | 0,50 | -4,47 | hemopoietic cell kinase |
| ROCK1 | 6093 | -20,77 | 154.25 | -2,29 | 1,03E-01 | 0,50 | -4,47 | Rho-associated coiled-coil containing protein kinase 1 |
| CNKSR2 | 22866 | -14,27 | 98.561 | -2,29 | 1,03E-01 | 0,50 | -4,47 | connector enhancer of kinase suppressor of Ras 2 |
| PIK3R5 | 23533 | -13,73 | 87.917 | -2,28 | 1,04E-01 | 0,50 | -4,47 | phosphoinositide-3-kinase regulatory subunit 5 |
| PDXK | 8566 | -258,90 | 1743.3 | -2,25 | 1,07E-01 | 0,51 | -4,48 | pyridoxal (pyridoxine vitamin B6) kinase |
| PSKH1 | 5681 | -14,50 | 112.47 | -2,25 | 1,07E-01 | 0,51 | -4,48 | protein serine kinase H1 |
| CLK3 | 1198 | -32,10 | 225.76 | -2,22 | 1,10E-01 | 0,52 | -4,48 | CDC-like kinase 3 |
| CDK2 | 1017 | 48,49 | 438.53 | 2,21 | 1,11E-01 | 0,52 | -4,48 | cyclin-dependent kinase 2 |
| GUK1 | 2987 | -51,93 | 1412.3 | -2,21 | 1,11E-01 | 0,52 | -4,48 | guanylate kinase 1 |
| AKAP13 | 11214 | -43,82 | 211.81 | -2,19 | 1,13E-01 | 0,52 | -4,48 | A kinase (PRKA) anchor protein 13 |
| PDK3 | 5165 | -17,70 | 152.51 | -2,17 | 1,15E-01 | 0,52 | -4,48 | pyruvate dehydrogenase kinase isozyme 3 |
| MAP3K2 | 10746 | -38,74 | 244.25 | -2,17 | 1,15E-01 | 0,52 | -4,48 | mitogen-activated protein kinase kinase kinase 2 |
| MAP2K3 | 5606 | -15,85 | 288.45 | -2,17 | 1,15E-01 | 0,52 | -4,48 | mitogen-activated protein kinase kinase 3 |
| BMP2K | 55589 | -17,93 | 153.68 | -2,16 | 1,16E-01 | 0,52 | -4,48 | BMP2 inducible kinase |
| PASK | 23178 | 12,50 | 116.21 | 2,15 | 1,18E-01 | 0,52 | -4,48 | PAS domain containing serine/threonine kinase |
| CDK6 | 1021 | -638,91 | 2020.8 | -2,11 | 1,22E-01 | 0,54 | -4,49 | cyclin-dependent kinase 6 |
| IPPK | 64768 | -14,43 | 108.13 | -2,10 | 1,24E-01 | 0,54 | -4,49 | inositol 1 3 4 5 6-pentakisphosphate 2-kinase |
| RIOK2 | 55781 | -129,33 | 505.97 | -2,09 | 1,25E-01 | 0,54 | -4,49 | RIO kinase 2 (yeast) |
| WDR83 | 84292 | -14,35 | 182.79 | -2,08 | 1,26E-01 | 0,54 | -4,49 | WD repeat domain 83 |
| MAPK6 | 5597 | -168,88 | 1150 | -2,08 | 1,26E-01 | 0,54 | -4,49 | mitogen-activated protein kinase 6 |
| AKAP8 | 10270 | -31,56 | 193.9 | -2,08 | 1,26E-01 | 0,54 | -4,49 | A kinase (PRKA) anchor protein 8 |
| NEK1 | 4750 | -23,59 | 192.36 | -2,06 | 1,28E-01 | 0,54 | -4,49 | NIMA (never in mitosis gene a)-related kinase 1 |
| CDKN2A | 1029 | -118,26 | 973.81 | -2,06 | 1,29E-01 | 0,54 | -4,49 | cyclin-dependent kinase inhibitor 2A (melanoma p16 inhibits CDK4) |
| NAGK | 55577 | -47,87 | 658.52 | -2,04 | 1,31E-01 | 0,54 | -4,49 | N-acetylglucosamine kinase |
| IRAK2 | 3656 | -32,63 | 147.52 | -2,02 | 1,33E-01 | 0,55 | -4,49 | interleukin-1 receptor-associated kinase 2 |
| GRK5 | 2869 | -14,66 | 137.34 | -2,00 | 1,36E-01 | 0,56 | -4,50 | G protein-coupled receptor kinase 5 |
| SIK3 | 23387 | -43,73 | 433.77 | -1,99 | 1,37E-01 | 0,56 | -4,50 | SIK family kinase 3 |
| ICK | 22858 | -16,02 | 146.65 | -1,98 | 1,38E-01 | 0,56 | -4,50 | intestinal cell (MAK-like) kinase |
| PIK3CG | 5294 | -14,78 | 87.401 | -1,97 | 1,41E-01 | 0,56 | -4,50 | phosphoinositide-3-kinase catalytic gamma polypeptide |
| MKNK1 | 8569 | 11,11 | 121.75 | 1,94 | 1,44E-01 | 0,57 | -4,50 | MAP kinase interacting serine/threonine kinase 1 |
| FASTKD3 | 79072 | -51,57 | 287.63 | -1,94 | 1,45E-01 | 0,57 | -4,50 | FAST kinase domains 3 |
| PFKFB3 | 5209 | -110,49 | 961.4 | -1,93 | 1,46E-01 | 0,57 | -4,50 | 6-phosphofructo-2-kinase/fructose-2 6-biphosphatase 3 |
| CDK5R1 | 8851 | -12,93 | 115.1 | -1,92 | 1,47E-01 | 0,57 | -4,50 | cyclin-dependent kinase 5 regulatory subunit 1 (p35) |
| LRGUK | 136332 | 19,05 | 87.457 | 1,92 | 1,48E-01 | 0,57 | -4,50 | leucine-rich repeats and guanylate kinase domain containing |
| CDK12 | 51755 | -17,26 | 152.9 | -1,90 | 1,50E-01 | 0,57 | -4,50 | cyclin-dependent kinase 12 |
| GIT2 | 9815 | -12,01 | 126.65 | -1,90 | 1,50E-01 | 0,57 | -4,50 | G protein-coupled receptor kinase interacting ArfGAP 2 |
| FASTKD2 | 22868 | -20,53 | 254.52 | -1,90 | 1,50E-01 | 0,57 | -4,50 | FAST kinase domains 2 |
| AKAP10 | 11216 | -22,24 | 144.64 | -1,86 | 1,57E-01 | 0,58 | -4,51 | A kinase (PRKA) anchor protein 10 |
| RPS6KA2 | 6196 | 24,39 | 227.02 | 1,85 | 1,59E-01 | 0,58 | -4,51 | ribosomal protein S6 kinase 90kDa polypeptide 2 |
| SIK1 | 150094 | -20,02 | 164.71 | -1,85 | 1,59E-01 | 0,58 | -4,51 | salt-inducible kinase 1 |
| MOB4 | 25843 | -108,71 | 754.26 | -1,85 | 1,59E-01 | 0,58 | -4,51 | MOB family member 4 phocein |
| SYK | 6850 | 101,59 | 528.03 | 1,84 | 1,60E-01 | 0,58 | -4,51 | spleen tyrosine kinase |
| PIP4K2A | 5305 | -35,23 | 225.69 | -1,83 | 1,61E-01 | 0,58 | -4,51 | phosphatidylinositol-5-phosphate 4-kinase type II alpha |
| PLK2 | 10769 | -96,66 | 665.5 | -1,83 | 1,62E-01 | 0,58 | -4,51 | polo-like kinase 2 |
| CAMKV | 79012 | -11,98 | 120.9 | -1,82 | 1,63E-01 | 0,58 | -4,51 | CaM kinase-like vesicle-associated |
| STK11IP | 114790 | -15,04 | 144.22 | -1,82 | 1,63E-01 | 0,58 | -4,51 | serine/threonine kinase 11 interacting protein |
| HKDC1 | 80201 | -15,22 | 258.16 | -1,81 | 1,65E-01 | 0,58 | -4,51 | hexokinase domain containing 1 |
| MAP3K4 | 4216 | -13,63 | 175.19 | -1,81 | 1,65E-01 | 0,58 | -4,51 | mitogen-activated protein kinase kinase kinase 4 |
| PRKX | 5613 | 21,61 | 215.73 | 1,80 | 1,66E-01 | 0,58 | -4,51 | protein kinase X-linked |
| MARK3 | 4140 | 25,45 | 188.5 | 1,80 | 1,67E-01 | 0,58 | -4,51 | MAP/microtubule affinity-regulating kinase 3 |
| MAP2K6 | 5608 | 16,73 | 113.75 | 1,79 | 1,68E-01 | 0,58 | -4,51 | mitogen-activated protein kinase kinase 6 |
| ALK | 238 | -36,38 | 177.21 | -1,79 | 1,69E-01 | 0,58 | -4,51 | anaplastic lymphoma receptor tyrosine kinase |
| PACSIN2 | 11252 | -34,48 | 543.78 | -1,78 | 1,70E-01 | 0,58 | -4,51 | protein kinase C and casein kinase substrate in neurons 2 |
| IRAK1 | 3654 | 58,74 | 1985.1 | 1,78 | 1,70E-01 | 0,58 | -4,52 | interleukin-1 receptor-associated kinase 1 |
| IP6K2 | 51447 | -23,54 | 244.82 | -1,75 | 1,75E-01 | 0,58 | -4,52 | inositol hexakisphosphate kinase 2 |
| ABL1 | 25 | -21,48 | 234.1 | -1,74 | 1,77E-01 | 0,58 | -4,52 | c-abl oncogene 1 non-receptor tyrosine kinase |
| PI4K2B | 55300 | -24,56 | 235.82 | -1,74 | 1,77E-01 | 0,58 | -4,52 | phosphatidylinositol 4-kinase type 2 beta |
| PFKFB4 | 5210 | 73,91 | 370.18 | 1,73 | 1,79E-01 | 0,58 | -4,52 | 6-phosphofructo-2-kinase/fructose-2 6-biphosphatase 4 |
| PFKP | 5214 | 133,08 | 847.02 | 1,72 | 1,80E-01 | 0,58 | -4,52 | phosphofructokinase platelet |
| PRKCDBP | 112464 | -15,16 | 102.73 | -1,72 | 1,80E-01 | 0,58 | -4,52 | protein kinase C delta binding protein |
| BMPR2 | 659 | -29,85 | 242.81 | -1,72 | 1,81E-01 | 0,58 | -4,52 | bone morphogenetic protein receptor type II (serine/threonine kinase) |
| IKBKG | 8517 | -58,52 | 698.5 | -1,72 | 1,81E-01 | 0,58 | -4,52 | inhibitor of kappa light polypeptide gene enhancer in B-cells kinase gamma |
| ZAK | 51776 | 22,04 | 250.98 | 1,71 | 1,83E-01 | 0,59 | -4,52 | sterile alpha motif and leucine zipper containing kinase AZK |
| MAPK8 | 5599 | -11,85 | 107.84 | -1,69 | 1,87E-01 | 0,60 | -4,52 | mitogen-activated protein kinase 8 |
| KDR | 3791 | -9,79 | 87.632 | -1,68 | 1,89E-01 | 0,60 | -4,52 | kinase insert domain receptor (a type III receptor tyrosine kinase) |
| TBK1 | 29110 | -44,85 | 503.84 | -1,66 | 1,93E-01 | 0,61 | -4,53 | TANK-binding kinase 1 |
| PIP4K2C | 79837 | -61,59 | 424.69 | -1,65 | 1,93E-01 | 0,61 | -4,53 | phosphatidylinositol-5-phosphate 4-kinase type II gamma |
| MOB3C | 148932 | -10,28 | 139.87 | -1,64 | 1,96E-01 | 0,61 | -4,53 | MOB kinase activator 3C |
| SBK1 | 388228 | 32,80 | 207.91 | 1,63 | 1,98E-01 | 0,61 | -4,53 | SH3-binding domain kinase 1 |
| STK38 | 11329 | -53,35 | 510.5 | -1,63 | 1,99E-01 | 0,61 | -4,53 | serine/threonine kinase 38 |
| MAPK1 | 5594 | -9,99 | 240.1 | -1,61 | 2,02E-01 | 0,62 | -4,53 | mitogen-activated protein kinase 1 |
| PRKAA1 | 5562 | -78,85 | 628.07 | -1,61 | 2,03E-01 | 0,62 | -4,53 | protein kinase AMP-activated alpha 1 catalytic subunit |
| PIK3C3 | 5289 | -22,85 | 184.51 | -1,60 | 2,05E-01 | 0,62 | -4,53 | phosphoinositide-3-kinase class 3 |
| FASTKD5 | 60493 | -74,24 | 549.92 | -1,60 | 2,06E-01 | 0,62 | -4,53 | FAST kinase domains 5 |
| DGKG | 1608 | 11,01 | 96.695 | 1,59 | 2,07E-01 | 0,62 | -4,53 | diacylglycerol kinase gamma 90kDa |
| NUAK2 | 81788 | 26,43 | 169.39 | 1,58 | 2,09E-01 | 0,62 | -4,53 | NUAK family SNF1-like kinase 2 |
| DCAKD | 79877 | 31,97 | 636.53 | 1,56 | 2,12E-01 | 0,63 | -4,54 | dephospho-CoA kinase domain containing |
| GK | 2710 | 9,56 | 118.46 | 1,56 | 2,12E-01 | 0,63 | -4,54 | glycerol kinase |
| PDK1 | 5163 | -9,03 | 91.134 | -1,55 | 2,15E-01 | 0,63 | -4,54 | pyruvate dehydrogenase kinase isozyme 1 |
| AK4 | 205 | -47,25 | 618.89 | -1,55 | 2,15E-01 | 0,63 | -4,54 | adenylate kinase 4 |
| CHKA | 1119 | -8,86 | 404.03 | -1,55 | 2,15E-01 | 0,63 | -4,54 | choline kinase alpha |
| SRPK1 | 6732 | -140,12 | 1320.3 | -1,53 | 2,20E-01 | 0,63 | -4,54 | SRSF protein kinase 1 |
| CALM3 | 808 | -107,02 | 5271 | -1,53 | 2,20E-01 | 0,63 | -4,54 | calmodulin 3 (phosphorylase kinase delta) |
| PLK3 | 1263 | -9,33 | 94.838 | -1,53 | 2,20E-01 | 0,63 | -4,54 | polo-like kinase 3 |
| AKAP12 | 9590 | -33,59 | 281.46 | -1,53 | 2,20E-01 | 0,63 | -4,54 | A kinase (PRKA) anchor protein 12 |
| PTK7 | 5754 | -22,20 | 280.28 | -1,53 | 2,21E-01 | 0,63 | -4,54 | PTK7 protein tyrosine kinase 7 |
| ULK2 | 9706 | -11,07 | 111.76 | -1,52 | 2,22E-01 | 0,63 | -4,54 | unc-51-like kinase 2 (C. elegans) |
| DYRK4 | 8798 | -42,53 | 313.45 | -1,51 | 2,24E-01 | 0,63 | -4,54 | dual-specificity tyrosine-(Y)-phosphorylation regulated kinase 4 |
| TK1 | 7083 | 234,00 | 1581 | 1,50 | 2,27E-01 | 0,63 | -4,54 | thymidine kinase 1 soluble |
| CDK20 | 23552 | -14,03 | 165.58 | -1,50 | 2,27E-01 | 0,63 | -4,54 | cyclin-dependent kinase 20 |
| NUCKS1 | 64710 | 302,48 | 6017.4 | 1,49 | 2,29E-01 | 0,63 | -4,54 | nuclear casein kinase and cyclin-dependent kinase substrate 1 |
| PFKM | 5213 | -63,07 | 473.84 | -1,49 | 2,30E-01 | 0,63 | -4,54 | phosphofructokinase muscle |
| MAPKAPK3 | 7867 | -162,12 | 1579.6 | -1,48 | 2,31E-01 | 0,63 | -4,54 | mitogen-activated protein kinase-activated protein kinase 3 |
| WNK2 | 65268 | -9,13 | 86.719 | -1,48 | 2,32E-01 | 0,63 | -4,54 | WNK lysine deficient protein kinase 2 |
| PLK1 | 5347 | -27,22 | 237.07 | -1,48 | 2,32E-01 | 0,63 | -4,54 | polo-like kinase 1 |
| PAK2 | 5062 | -55,49 | 604.95 | -1,46 | 2,36E-01 | 0,64 | -4,55 | p21 protein (Cdc42/Rac)-activated kinase 2 |
| PINK1 | 65018 | -17,98 | 192.18 | -1,46 | 2,37E-01 | 0,64 | -4,55 | PTEN induced putative kinase 1 |
| STK17B | 9262 | -11,59 | 195.95 | -1,46 | 2,38E-01 | 0,64 | -4,55 | serine/threonine kinase 17b |
| MYLK | 4638 | -18,00 | 166.28 | -1,45 | 2,39E-01 | 0,64 | -4,55 | myosin light chain kinase |
| RIOK3 | 8780 | -64,82 | 647.77 | -1,43 | 2,45E-01 | 0,64 | -4,55 | RIO kinase 3 (yeast) |
| NADK | 65220 | 14,16 | 177.95 | 1,42 | 2,46E-01 | 0,64 | -4,55 | NAD kinase |
| PRKG2 | 5593 | -8,20 | 90.157 | -1,42 | 2,48E-01 | 0,64 | -4,55 | protein kinase cGMP-dependent type II |
| TXK | 7294 | -8,40 | 83.163 | -1,42 | 2,48E-01 | 0,64 | -4,55 | TXK tyrosine kinase |
| STRADB | 55437 | 17,22 | 220.21 | 1,42 | 2,49E-01 | 0,64 | -4,55 | STE20-related kinase adaptor beta |
| PDPK1 | 5170 | -13,37 | 180.96 | -1,42 | 2,49E-01 | 0,64 | -4,55 | 3-phosphoinositide dependent protein kinase-1 |
| STK3 | 6788 | -59,53 | 751.15 | -1,41 | 2,51E-01 | 0,64 | -4,55 | serine/threonine kinase 3 |
| MST4 | 51765 | -17,05 | 818.51 | -1,41 | 2,51E-01 | 0,64 | -4,55 | serine/threonine protein kinase MST4 |
| ADCK2 | 90956 | 41,29 | 509.13 | 1,40 | 2,52E-01 | 0,64 | -4,55 | aarF domain containing kinase 2 |
| AKAP1 | 8165 | -20,99 | 219.64 | -1,40 | 2,54E-01 | 0,64 | -4,55 | A kinase (PRKA) anchor protein 1 |
| TSSK4 | 283629 | -8,98 | 86.097 | -1,39 | 2,57E-01 | 0,65 | -4,56 | testis-specific serine kinase 4 |
| PIK3R2 | 5296 | 152,46 | 1579.7 | 1,38 | 2,59E-01 | 0,65 | -4,56 | phosphoinositide-3-kinase regulatory subunit 2 (beta) |
| LIMK2 | 3985 | 11,18 | 138.31 | 1,38 | 2,59E-01 | 0,65 | -4,56 | LIM domain kinase 2 |
| MLKL | 197259 | -10,89 | 183.31 | -1,37 | 2,60E-01 | 0,65 | -4,56 | mixed lineage kinase domain-like |
| FLT3LG | 2323 | -8,35 | 93.09 | -1,37 | 2,60E-01 | 0,65 | -4,56 | fms-related tyrosine kinase 3 ligand |
| NME7 | 29922 | -61,86 | 545.13 | -1,36 | 2,64E-01 | 0,65 | -4,56 | non-metastatic cells 7 protein expressed in (nucleoside-diphosphate kinase) |
| MAP3K6 | 9064 | -48,93 | 484.94 | -1,35 | 2,66E-01 | 0,65 | -4,56 | mitogen-activated protein kinase kinase kinase 6 |
| LIMK1 | 3984 | -9,34 | 93.955 | -1,35 | 2,66E-01 | 0,65 | -4,56 | LIM domain kinase 1 |
| PIP4K2B | 8396 | -9,79 | 114.87 | -1,35 | 2,66E-01 | 0,65 | -4,56 | phosphatidylinositol-5-phosphate 4-kinase type II beta |
| NEK5 | 341676 | 7,82 | 75.2 | 1,33 | 2,74E-01 | 0,66 | -4,56 | NIMA (never in mitosis gene a)-related kinase 5 |
| MYLK4 | 340156 | -8,21 | 86.361 | -1,32 | 2,77E-01 | 0,67 | -4,56 | myosin light chain kinase family member 4 |
| DAPK3 | 1613 | -13,20 | 129.59 | -1,31 | 2,80E-01 | 0,67 | -4,56 | death-associated protein kinase 3 |
| PRKCSH | 5589 | 15,41 | 549.1 | 1,30 | 2,83E-01 | 0,67 | -4,57 | protein kinase C substrate 80K-H |
| CDK2AP2 | 10263 | 26,69 | 283.32 | 1,30 | 2,83E-01 | 0,67 | -4,57 | cyclin-dependent kinase 2 associated protein 2 |
| TTK | 7272 | -47,48 | 876.26 | -1,29 | 2,85E-01 | 0,67 | -4,57 | TTK protein kinase |
| FN3KRP | 79672 | 54,87 | 647.69 | 1,28 | 2,86E-01 | 0,67 | -4,57 | fructosamine 3 kinase related protein |
| PACSIN1 | 29993 | -12,91 | 130.12 | -1,28 | 2,87E-01 | 0,67 | -4,57 | protein kinase C and casein kinase substrate in neurons 1 |
| RPS6KA3 | 6197 | -9,57 | 134.59 | -1,28 | 2,88E-01 | 0,67 | -4,57 | ribosomal protein S6 kinase 90kDa polypeptide 3 |
| PRKCZ | 5590 | -27,99 | 313.1 | -1,28 | 2,88E-01 | 0,67 | -4,57 | protein kinase C zeta |
| DYRK3 | 8444 | -8,10 | 113.41 | -1,28 | 2,89E-01 | 0,67 | -4,57 | dual-specificity tyrosine-(Y)-phosphorylation regulated kinase 3 |
| PICK1 | 9463 | 15,81 | 122.17 | 1,27 | 2,90E-01 | 0,67 | -4,57 | protein interacting with PRKCA 1 |
| CDK4 | 1019 | 268,62 | 3055.6 | 1,27 | 2,90E-01 | 0,67 | -4,57 | cyclin-dependent kinase 4 |
| DGKD | 8527 | -9,01 | 99.492 | -1,27 | 2,90E-01 | 0,67 | -4,57 | diacylglycerol kinase delta 130kDa |
| STK25 | 10494 | 29,37 | 720.39 | 1,26 | 2,93E-01 | 0,67 | -4,57 | serine/threonine kinase 25 |
| NEK11 | 79858 | -8,13 | 158.65 | -1,26 | 2,94E-01 | 0,67 | -4,57 | NIMA (never in mitosis gene a)- related kinase 11 |
| DCLK1 | 9201 | -7,78 | 101.26 | -1,26 | 2,94E-01 | 0,67 | -4,57 | doublecortin-like kinase 1 |
| CDK18 | 5129 | -8,15 | 95.89 | -1,25 | 2,96E-01 | 0,67 | -4,57 | cyclin-dependent kinase 18 |
| STRADA | 92335 | 14,24 | 201.73 | 1,25 | 2,96E-01 | 0,67 | -4,57 | STE20-related kinase adaptor alpha |
| MAP3K10 | 4294 | -22,96 | 191.92 | -1,25 | 2,98E-01 | 0,67 | -4,57 | mitogen-activated protein kinase kinase kinase 10 |
| DAPK2 | 23604 | -12,91 | 96.013 | -1,24 | 3,00E-01 | 0,67 | -4,57 | death-associated protein kinase 2 |
| SPHK1 | 8877 | -7,10 | 89.101 | -1,24 | 3,01E-01 | 0,67 | -4,57 | sphingosine kinase 1 |
| STK38L | 23012 | -8,16 | 109.9 | -1,23 | 3,02E-01 | 0,67 | -4,57 | serine/threonine kinase 38 like |
| JAKMIP2 | 9832 | -10,67 | 86.57 | -1,23 | 3,03E-01 | 0,67 | -4,57 | janus kinase and microtubule interacting protein 2 |
| AK2 | 204 | 93,46 | 2345.5 | 1,22 | 3,07E-01 | 0,67 | -4,57 | adenylate kinase 2 |
| TSSK6 | 83983 | 7,29 | 94.73 | 1,21 | 3,09E-01 | 0,67 | -4,57 | testis-specific serine kinase 6 |
| MYLK2 | 85366 | 9,03 | 111.77 | 1,20 | 3,14E-01 | 0,68 | -4,58 | myosin light chain kinase 2 |
| RPS6KA4 | 8986 | -27,91 | 434.28 | -1,20 | 3,15E-01 | 0,68 | -4,58 | ribosomal protein S6 kinase 90kDa polypeptide 4 |
| TYK2 | 7297 | -25,29 | 795.73 | -1,19 | 3,16E-01 | 0,68 | -4,58 | tyrosine kinase 2 |
| ADCK5 | 203054 | -8,91 | 115.49 | -1,19 | 3,16E-01 | 0,68 | -4,58 | aarF domain containing kinase 5 |
| ETNK1 | 55500 | -8,71 | 169.17 | -1,18 | 3,21E-01 | 0,69 | -4,58 | ethanolamine kinase 1 |
| AKAP11 | 11215 | -10,17 | 171.65 | -1,18 | 3,21E-01 | 0,69 | -4,58 | A kinase (PRKA) anchor protein 11 |
| PRKCQ | 5588 | 8,66 | 153.96 | 1,17 | 3,23E-01 | 0,69 | -4,58 | protein kinase C theta |
| WNK3 | 65267 | -7,16 | 97.942 | -1,16 | 3,26E-01 | 0,69 | -4,58 | WNK lysine deficient protein kinase 3 |
| CIT | 11113 | -7,77 | 89.185 | -1,15 | 3,29E-01 | 0,69 | -4,58 | citron (rho-interacting serine/threonine kinase 21) |
| GALK2 | 2585 | -7,42 | 126.75 | -1,15 | 3,31E-01 | 0,69 | -4,58 | galactokinase 2 |
| PRKY | 5616 | -8,08 | 91.856 | -1,15 | 3,31E-01 | 0,69 | -4,58 | protein kinase Y-linked pseudogene |
| CDK2AP1 | 8099 | 406,06 | 5954.4 | 1,15 | 3,32E-01 | 0,69 | -4,58 | cyclin-dependent kinase 2 associated protein 1 |
| CDKN2D | 1032 | 7,02 | 175.56 | 1,14 | 3,33E-01 | 0,69 | -4,58 | cyclin-dependent kinase inhibitor 2D (p19 inhibits CDK4) |
| JAK1 | 3716 | 30,40 | 346.94 | 1,14 | 3,33E-01 | 0,69 | -4,58 | Janus kinase 1 |
| ADRBK2 | 157 | -8,25 | 89.676 | -1,14 | 3,35E-01 | 0,69 | -4,58 | adrenergic beta receptor kinase 2 |
| STK17A | 9263 | -10,00 | 116.62 | -1,14 | 3,36E-01 | 0,69 | -4,58 | serine/threonine kinase 17a |
| PFKL | 5211 | 6,82 | 178.69 | 1,14 | 3,36E-01 | 0,69 | -4,58 | phosphofructokinase liver |
| PRKD1 | 5587 | -13,14 | 133.76 | -1,13 | 3,37E-01 | 0,69 | -4,58 | protein kinase D1 |
| CSNK1A1P1 | 161635 | 9,53 | 90.098 | 1,13 | 3,40E-01 | 0,69 | -4,59 | casein kinase 1 alpha 1 pseudogene 1 |
| TSSK2 | 23617 | -6,57 | 86.304 | -1,12 | 3,43E-01 | 0,69 | -4,59 | testis-specific serine kinase 2 |
| AK3 | 50808 | 36,52 | 1350.1 | 1,10 | 3,49E-01 | 0,70 | -4,59 | adenylate kinase 3 |
| STK24 | 8428 | -29,05 | 789.18 | -1,10 | 3,49E-01 | 0,70 | -4,59 | serine/threonine kinase 24 |
| AKAP5 | 9495 | -6,87 | 84.999 | -1,09 | 3,53E-01 | 0,71 | -4,59 | A kinase (PRKA) anchor protein 5 |
| PRKRIR | 5612 | -361,93 | 4582.3 | -1,07 | 3,59E-01 | 0,71 | -4,59 | protein-kinase interferon-inducible double stranded RNA dependent inhibitor repressor of (P58 repressor) |
| PANK1 | 53354 | 9,23 | 179.03 | 1,07 | 3,59E-01 | 0,71 | -4,59 | pantothenate kinase 1 |
| CKS1B | 1163 | 292,88 | 3350 | 1,07 | 3,62E-01 | 0,71 | -4,59 | CDC28 protein kinase regulatory subunit 1B |
| MAP4K2 | 5871 | 100,55 | 1549.6 | 1,06 | 3,63E-01 | 0,71 | -4,59 | mitogen-activated protein kinase kinase kinase kinase 2 |
| MAST4 | 375449 | -6,88 | 93.156 | -1,06 | 3,63E-01 | 0,71 | -4,59 | microtubule associated serine/threonine kinase family member 4 |
| PDK4 | 5166 | 7,15 | 84.825 | 1,06 | 3,63E-01 | 0,71 | -4,59 | pyruvate dehydrogenase kinase isozyme 4 |
| MOB1B | 92597 | -16,16 | 165.81 | -1,06 | 3,66E-01 | 0,71 | -4,59 | MOB kinase activator 1B |
| PIP5K1B | 8395 | -7,54 | 92.221 | -1,06 | 3,66E-01 | 0,71 | -4,59 | phosphatidylinositol-4-phosphate 5-kinase type I beta |
| MVK | 4598 | -9,44 | 116.86 | -1,05 | 3,69E-01 | 0,72 | -4,59 | mevalonate kinase |
| LCK | 3932 | -6,48 | 92.867 | -1,04 | 3,73E-01 | 0,72 | -4,60 | lymphocyte-specific protein tyrosine kinase |
| ITPK1 | 3705 | 6,53 | 151.44 | 1,04 | 3,74E-01 | 0,72 | -4,60 | inositol-tetrakisphosphate 1-kinase |
| DGKK | 139189 | 6,90 | 80.971 | 1,03 | 3,76E-01 | 0,72 | -4,60 | diacylglycerol kinase kappa |
| CDK5R2 | 8941 | 8,34 | 97.287 | 1,03 | 3,77E-01 | 0,72 | -4,60 | cyclin-dependent kinase 5 regulatory subunit 2 (p39) |
| GRK4 | 2868 | -7,00 | 83.016 | -1,03 | 3,77E-01 | 0,72 | -4,60 | G protein-coupled receptor kinase 4 |
| MAPK11 | 5600 | -6,15 | 93.01 | -1,02 | 3,80E-01 | 0,72 | -4,60 | mitogen-activated protein kinase 11 |
| LAMTOR3 | 8649 | -89,17 | 1022 | -1,02 | 3,81E-01 | 0,72 | -4,60 | late endosomal/lysosomal adaptor MAPK and MTOR activator 3 |
| IBTK | 25998 | -34,42 | 631.82 | -1,02 | 3,81E-01 | 0,72 | -4,60 | inhibitor of Bruton agammaglobulinemia tyrosine kinase |
| BRSK2 | 9024 | 5,80 | 93.115 | 1,00 | 3,87E-01 | 0,73 | -4,60 | BR serine/threonine kinase 2 |
| PXK | 54899 | -9,16 | 117.2 | -1,00 | 3,90E-01 | 0,73 | -4,60 | PX domain containing serine/threonine kinase |
| AKAP3 | 10566 | -9,80 | 92.698 | -0,99 | 3,94E-01 | 0,74 | -4,60 | A kinase (PRKA) anchor protein 3 |
| CDKN2C | 1031 | 6,83 | 106.45 | 0,98 | 3,95E-01 | 0,74 | -4,60 | cyclin-dependent kinase inhibitor 2C (p18 inhibits CDK4) |
| PTK2B | 2185 | -7,74 | 110.23 | -0,98 | 3,97E-01 | 0,74 | -4,60 | PTK2B protein tyrosine kinase 2 beta |
| GKAP1 | 80318 | 5,75 | 100.05 | 0,97 | 4,00E-01 | 0,74 | -4,60 | G kinase anchoring protein 1 |
| MARK2 | 2011 | -6,34 | 123.51 | -0,97 | 4,00E-01 | 0,74 | -4,60 | MAP/microtubule affinity-regulating kinase 2 |
| PRKACB | 5567 | -5,74 | 92.495 | -0,97 | 4,01E-01 | 0,74 | -4,60 | protein kinase cAMP-dependent catalytic beta |
| MAPK8IP2 | 23542 | -5,53 | 89.006 | -0,97 | 4,03E-01 | 0,74 | -4,60 | mitogen-activated protein kinase 8 interacting protein 2 |
| DTYMK | 1841 | 5,62 | 95.206 | 0,96 | 4,04E-01 | 0,74 | -4,60 | deoxythymidylate kinase (thymidylate kinase) |
| MAPK13 | 5603 | -15,00 | 347.91 | -0,95 | 4,08E-01 | 0,74 | -4,61 | mitogen-activated protein kinase 13 |
| TIE1 | 7075 | 5,48 | 86.665 | 0,95 | 4,09E-01 | 0,74 | -4,61 | tyrosine kinase with immunoglobulin-like and EGF-like domains 1 |
| DCK | 1633 | -19,12 | 326.43 | -0,95 | 4,12E-01 | 0,74 | -4,61 | deoxycytidine kinase |
| SKP1 | 6500 | -48,85 | 819.48 | -0,94 | 4,14E-01 | 0,75 | -4,61 | S-phase kinase-associated protein 1 |
| SRMS | 6725 | -5,45 | 79.881 | -0,94 | 4,16E-01 | 0,75 | -4,61 | src-related kinase lacking C-terminal regulatory tyrosine and N-terminal myristylation sites |
| STK35 | 140901 | -7,56 | 137.08 | -0,93 | 4,19E-01 | 0,75 | -4,61 | serine/threonine kinase 35 |
| ADCK4 | 79934 | 9,57 | 133.06 | 0,92 | 4,22E-01 | 0,75 | -4,61 | aarF domain containing kinase 4 |
| AK5 | 26289 | -5,95 | 85.88 | -0,92 | 4,22E-01 | 0,75 | -4,61 | adenylate kinase 5 |
| TEK | 7010 | -6,44 | 87.953 | -0,92 | 4,23E-01 | 0,75 | -4,61 | TEK tyrosine kinase endothelial |
| MAPK12 | 6300 | 5,85 | 106.73 | 0,92 | 4,25E-01 | 0,75 | -4,61 | mitogen-activated protein kinase 12 |
| PKMYT1 | 9088 | 6,56 | 139.28 | 0,91 | 4,26E-01 | 0,75 | -4,61 | protein kinase membrane associated tyrosine/threonine 1 |
| CDK17 | 5128 | -9,88 | 104.75 | -0,91 | 4,27E-01 | 0,75 | -4,61 | cyclin-dependent kinase 17 |
| MAP3K11 | 4296 | 9,78 | 177.27 | 0,91 | 4,28E-01 | 0,75 | -4,61 | mitogen-activated protein kinase kinase kinase 11 |
| RYK | 6259 | -140,35 | 1903.6 | -0,91 | 4,29E-01 | 0,75 | -4,61 | receptor-like tyrosine kinase |
| CMPK1 | 51727 | -36,68 | 693.04 | -0,90 | 4,32E-01 | 0,75 | -4,61 | cytidine monophosphate (UMP-CMP) kinase 1 cytosolic |
| BCKDK | 10295 | 34,06 | 1083.5 | 0,89 | 4,35E-01 | 0,75 | -4,61 | branched chain ketoacid dehydrogenase kinase |
| PFKFB2 | 5208 | -6,26 | 108.37 | -0,89 | 4,36E-01 | 0,75 | -4,61 | 6-phosphofructo-2-kinase/fructose-2 6-biphosphatase 2 |
| SGK494 | 124923 | -7,06 | 104.23 | -0,88 | 4,39E-01 | 0,76 | -4,61 | uncharacterized serine/threonine-protein kinase SgK494 |
| CSK | 1445 | -23,00 | 566.63 | -0,87 | 4,44E-01 | 0,76 | -4,61 | c-src tyrosine kinase |
| CSNK2B | 1460 | -103,94 | 2196.4 | -0,87 | 4,47E-01 | 0,76 | -4,62 | casein kinase 2 beta polypeptide |
| CAMK1D | 57118 | 6,55 | 130.69 | 0,87 | 4,47E-01 | 0,76 | -4,62 | calcium/calmodulin-dependent protein kinase ID |
| TYRO3P | 7302 | -5,19 | 76.758 | -0,86 | 4,49E-01 | 0,76 | -4,62 | TYRO3P protein tyrosine kinase pseudogene |
| NTRK3 | 4916 | -6,09 | 85.834 | -0,86 | 4,49E-01 | 0,76 | -4,62 | neurotrophic tyrosine kinase receptor type 3 |
| CLK4 | 57396 | -5,19 | 117.48 | -0,86 | 4,49E-01 | 0,76 | -4,62 | CDC-like kinase 4 |
| CDC42BPB | 9578 | -32,12 | 502.47 | -0,85 | 4,57E-01 | 0,77 | -4,62 | CDC42 binding protein kinase beta (DMPK-like) |
| DGKE | 8526 | -9,94 | 122.57 | -0,83 | 4,63E-01 | 0,78 | -4,62 | diacylglycerol kinase epsilon 64kDa |
| MARK1 | 4139 | -8,80 | 111.9 | -0,83 | 4,64E-01 | 0,78 | -4,62 | MAP/microtubule affinity-regulating kinase 1 |
| PRKCA | 5578 | 10,93 | 262.14 | 0,82 | 4,69E-01 | 0,78 | -4,62 | protein kinase C alpha |
| GAK | 2580 | -70,24 | 1698.6 | -0,82 | 4,71E-01 | 0,78 | -4,62 | cyclin G associated kinase |
| BLK | 640 | -4,71 | 77.251 | -0,82 | 4,72E-01 | 0,78 | -4,62 | B lymphoid tyrosine kinase |
| GLYCTK | 132158 | -6,20 | 129.25 | -0,81 | 4,75E-01 | 0,79 | -4,62 | glycerate kinase |
| PLK5 | 126520 | -4,66 | 83.103 | -0,81 | 4,75E-01 | 0,79 | -4,62 | polo-like kinase 5 |
| PAK4 | 10298 | 13,86 | 673.05 | 0,81 | 4,78E-01 | 0,79 | -4,62 | p21 protein (Cdc42/Rac)-activated kinase 4 |
| NLK | 51701 | -11,33 | 183.49 | -0,80 | 4,81E-01 | 0,79 | -4,62 | nemo-like kinase |
| ITK | 3702 | -6,02 | 88.252 | -0,80 | 4,82E-01 | 0,79 | -4,62 | IL2-inducible T-cell kinase |
| MOB3B | 79817 | -10,38 | 192.56 | -0,79 | 4,83E-01 | 0,79 | -4,62 | MOB kinase activator 3B |
| PAK6 | 56924 | 7,89 | 124.66 | 0,79 | 4,84E-01 | 0,79 | -4,62 | p21 protein (Cdc42/Rac)-activated kinase 6 |
| TPK1 | 27010 | -44,10 | 557.94 | -0,79 | 4,86E-01 | 0,79 | -4,62 | thiamin pyrophosphokinase 1 |
| CAMK2N1 | 55450 | 38,44 | 226.44 | 0,78 | 4,91E-01 | 0,79 | -4,63 | calcium/calmodulin-dependent protein kinase II inhibitor 1 |
| TTBK2 | 146057 | -5,67 | 83.616 | -0,78 | 4,92E-01 | 0,79 | -4,63 | tau tubulin kinase 2 |
| MELK | 9833 | 26,24 | 1435.2 | 0,77 | 4,95E-01 | 0,79 | -4,63 | maternal embryonic leucine zipper kinase |
| PRKCH | 5583 | -31,69 | 539.48 | -0,77 | 4,95E-01 | 0,79 | -4,63 | protein kinase C eta |
| RBKS | 64080 | -15,89 | 286.98 | -0,77 | 4,96E-01 | 0,79 | -4,63 | ribokinase |
| GNE | 10020 | -8,51 | 326.03 | -0,77 | 4,96E-01 | 0,79 | -4,63 | glucosamine (UDP-N-acetyl)-2-epimerase/N-acetylmannosamine kinase |
| ALPK3 | 57538 | -5,64 | 85.704 | -0,77 | 4,97E-01 | 0,79 | -4,63 | alpha-kinase 3 |
| PIP5K1C | 23396 | 6,11 | 152.99 | 0,76 | 4,99E-01 | 0,79 | -4,63 | phosphatidylinositol-4-phosphate 5-kinase type I gamma |
